# Increased expression of schizophrenia-associated gene C4 leads to hypoconnectivity of prefrontal cortex and reduced social interaction

**DOI:** 10.1371/journal.pbio.3000604

**Published:** 2020-01-14

**Authors:** Ashley L. Comer, Tushare Jinadasa, Balaji Sriram, Rhushikesh A. Phadke, Lisa N. Kretsge, Thanh P. H. Nguyen, Giovanna Antognetti, James P. Gilbert, Jungjoon Lee, Elena R. Newmark, Frances S. Hausmann, SaraAnn Rosenthal, Kevin Liu Kot, Yenyu Liu, William W. Yen, Borislav Dejanovic, Alberto Cruz-Martín

**Affiliations:** 1 Department of Biology, Boston University, Boston, Massachusetts, United States of America; 2 The Graduate Program for Neuroscience, Boston University, Boston, Massachusetts, United States of America; 3 Neurophotonics Center, Boston University, Boston, Massachusetts, United States of America; 4 Research and Early Development, Biogen, Cambridge, Massachusetts, United States of America; 5 Molecular Biology, Cell Biology and Biochemistry Program, Boston University, Boston, Massachusetts, United States of America; 6 Department of Biomedical Engineering, Boston University, Boston, Massachusetts, United States of America; 7 Biologics Drug Discovery, Biogen, Cambridge, Massachusetts, United States of America; 8 External Innovations and New Indications, Biogen, Cambridge, Massachusetts, United States of America; 9 Department of Biology, Connecticut College, New London, Connecticut, United States of America; 10 Biochemistry and Molecular Biology/Biotechnology Program, Boston University, Boston, Massachusetts, United States of America; 11 Stanley Center for Psychiatric Research, Broad Institute of MIT and Harvard, Cambridge, Massachusetts, United States of America; 12 Department Pharmacology and Experimental Therapeutics, Boston University, Boston, Massachusetts, United States of America; 13 Center for Systems Neuroscience, Boston University, Boston, Massachusetts, United States of America; Mount Sinai School of Medicine, UNITED STATES

## Abstract

Schizophrenia is a severe mental disorder with an unclear pathophysiology. Increased expression of the immune gene C4 has been linked to a greater risk of developing schizophrenia; however, it is not known whether C4 plays a causative role in this brain disorder. Using confocal imaging and whole-cell electrophysiology, we demonstrate that overexpression of C4 in mouse prefrontal cortex neurons leads to perturbations in dendritic spine development and hypoconnectivity, which mirror neuropathologies found in schizophrenia patients. We find evidence that microglia-mediated synaptic engulfment is enhanced with increased expression of C4. We also show that C4-dependent circuit dysfunction in the frontal cortex leads to decreased social interactions in juvenile and adult mice. These results demonstrate that increased expression of the schizophrenia-associated gene C4 causes aberrant circuit wiring in the developing prefrontal cortex and leads to deficits in juvenile and adult social behavior, suggesting that altered C4 expression contributes directly to schizophrenia pathogenesis.

## Abstract 2

Elevated expression of the gene encoding complement C4 is associated with an enhanced risk of schizophrenia, but the mechanism underlying this link is unclear. This study shows that overexpression of the C4 gene in mice leads to mis-wiring of the prefrontal cortex and deficits in social interactions.

## Introduction

Schizophrenia (SCZ) is a neurodevelopmental disorder characterized by disruptions in brain connectivity that lead to an array of symptoms including psychosis and deficits in cognition and social interactions [1,2]. Current treatment options are somewhat effective for treating psychosis but do not improve cognitive or social deficits, both of which contribute significantly to the long-term prognosis of people with SCZ [3–5]. Early social deficits are a feature of this disease and are often present even before the onset of psychosis [6]. Therefore, understanding the underlying mechanisms of early social deficits in SCZ could reveal a therapeutic window to alter disease progression in these individuals.

Among the many clinical traits of SCZ are deficits in social cognition such as social-cue perception and emotion regulation, which suggest dysfunction of the prefrontal cortex (PFC) [5]. Moreover, patients with SCZ exhibit abnormal activity in the PFC during affective face perception and cognitive reappraisal [7–9]. These results are not surprising given the well-established role of the PFC in social behaviors [10–12]. Although the mechanisms that cause social deficits in SCZ are unclear, evidence suggests that dysfunction in the PFC correlates with symptom onset and severity [13]. In fact, a neurological hallmark of SCZ is a reduction of gray matter in the PFC due to a loss of neuronal processes and synapses [14,15]. Therefore, it has been hypothesized that pathology in SCZ arises in part because of deficits in the pruning of cortical synapses, thus producing aberrant circuitry [14,15].

SCZ is highly heritable and genome-wide association studies have shown that the major histocompatibility complex (MHC) has the greatest genetic association with SCZ [16–19]. In particular, *C4*, which is located in the MHC locus, is associated with SCZ such that specific structural variants and mutations that cause increased expression of *C4* confer greater risk for this brain disorder [16]. Human *C4* (h*C4*) is encoded by two distinct genes, or isotypes, *C4A* and *C4B*, whereas mouse *C4* (m*C4*), encoded by a single gene (*C4b)*, contains an amino acid sequence that is approximately 80% similar to both h*C4* isotypes [20–22]. C4 is a member of the innate immunity complement cascade that forms a proteolytic protein cascade that clears debris, enhances inflammation, and tags pathogens for engulfment or destruction [23]. It has been shown that microglia, the brain’s resident macrophage, engulf neuronal and synaptic material dependent on the presence of complement proteins [24,25]. Microglia are the only cells in the brain that express the complement 3 receptor (CR3), which allows microglia to recognize and phagocytose material tagged by the complement cascade [24–26]. Microglia-mediated synaptic pruning is necessary for normal development of the brain but can also contribute to abnormal wiring of circuits and pathology when mis-regulated [16,27–30].

Despite significant progress in this field, it is still not clear how reciprocal interactions between neurons and microglia contribute to the maturation and refinement of developing cortical circuits [31–33]. Mouse studies investigating the role of C4 and other complement proteins in synaptic refinement have relied on loss-of-function manipulations [16,24,25,34], whereas SCZ is linked to increased C4 expression [16]. Additionally, previous studies have focused on the role of complement proteins in the visual system [16,24,25] rather than interrogating their role in more SCZ-relevant brain regions, such as the PFC. Thus, overall, it is not known how increased C4 expression impacts the development of cortical microcircuits, which are commonly altered in neurodevelopmental disorders [35,36].

Herein, we investigated the effects of C4 overexpression on developing networks in layer (L) 2/3 of the mouse medial prefrontal cortex (mPFC), a cortical region where gray-matter loss is most prominent in SCZ [14,15]. We show that C4 overexpression induced transient structural and functional changes to L2/3 excitatory neurons in the mPFC. Additionally, we interrogated the role of microglia in complement-induced wiring deficits in the mPFC by measuring microglia engulfment of synaptic material. Lastly, we examined the relationship between C4-induced PFC circuit dysfunction and abnormalities in rodent social behavior. Taken together, our results link C4 overexpression to neural and behavioral deficits relevant to SCZ and identify a critical window in which the trajectory of brain development might be able to be therapeutically targeted in SCZ.

## Results

### mC4 is expressed in neurons of the mPFC and can be overexpressed using in utero electroporation

**Fig 1:**
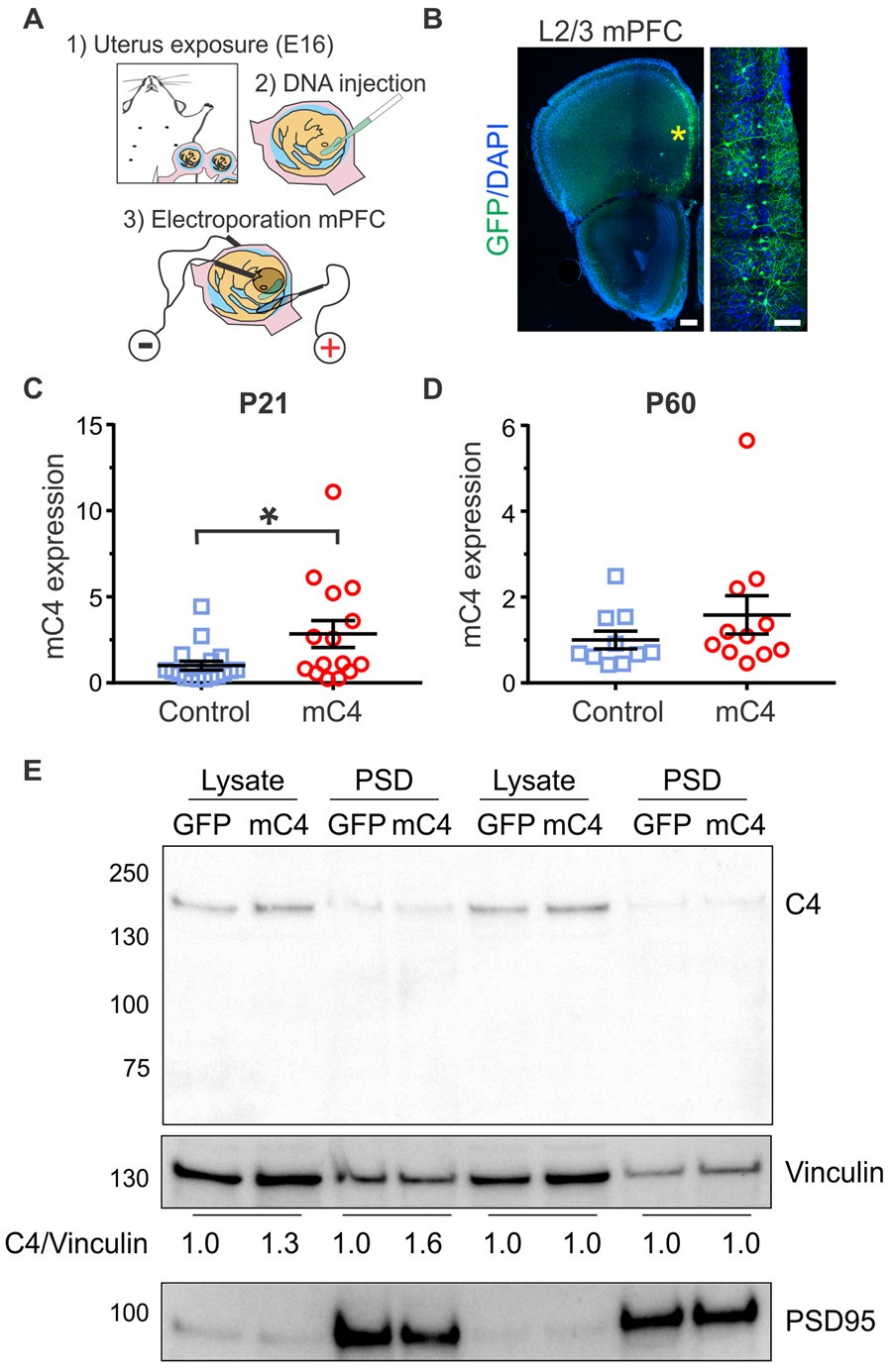
mC4 is expressed in neurons of the mPFC and can be overexpressed using IUE. (A) Diagram depicting IUE surgery performed in E16 dams. (B) Representative 20X confocal image of IUE with GFP targeted to L2/3 mPFC, the electroporated region. Yellow asterisk: L2/3 GFP+ neurons. Left panel scale bar = 250 μm. Right panel scale bar = 75 μm. (C) IUE increases mC4 expression by 2.84-fold compared to P21 control. *N* = 18 control mice. *N* = 15 mC4 mice. *t* test with Welch’s correction. *p* = 0.0392. (D) mC4 mRNA expression at P60 for control and mC4 conditions. *N* = 10 control mice. *N* = 11 mC4 mice. *t* test with Welch’s correction. *p* = 0.2564. (C-D) qPCR performed from the dissected electroporated region. Control: blue. mC4: red. Mean ± SEM. (E) Immunoblot (top) and quantification (bottom) of relative mC4 levels in total lysates and isolated PSD fractions from GFP or mC4 conditions from the electroporated region. Since C4 was expressed at relatively low levels, it could only be detected when brains were pooled. Lysates and PSDs from the electroporated region were prepared from the individual mice and pooled for the western blot analysis. *N* = 7 mice per group for P21 (left 4 lanes). *N* = 4 mice per group for P60 (right 4 lanes). Vinculin was used as loading control for lysates and PSD fractions. PSD-95 immunoblot served as a control for successful isolation of PSD fractions. For underlying data, see https://osf.io/7em3s/?view_only=0e7ffde4ebd344dc83af83b5a605c451. E, embryonic day; GFP, green fluorescent protein; IUE, in utero electroporation; L2/3, layer 2/3; mC4, mouse C4; mPFC, medial prefrontal cortex; P, postnatal day; PSD, postsynaptic density; qPCR, quantitative PCR.

Using multiplex in situ hybridization (M-FISH), we showed that PFC neurons in postnatal day (P) 30 control mice express low levels of C4b transcript (S1A Fig), which was not present in tissue from *C4b* knock-out (*C4* KO) mice (S1B Fig). To target L2/3 mPFC pyramidal neurons for *C4* overexpression, we used in utero electroporation (IUE) in mice at embryonic day (E) 16 (Fig 1A and 1B) [37]. To overexpress C4, we performed IUE using plasmids with the CAG promoter to coexpress green fluorescent protein (GFP) and m*C4b* (mC4 condition). Then, we used M-FISH at P21 to quantify the extent of C4 overexpression achieved. Within the same coronal section and cortical layer of the mPFC, we identified excitatory L2/3 neurons that were either transfected (GFP+/calcium/calmodulin-dependent protein kinase type II subunit alpha [CaMKIIα]+) or untransfected (GFP−/CaMKIIα+) and compared the percent of C4 mRNA+ cell body areas (S1C Fig). This allowed us to quantify both endogenous expression of mC4 in pyramidal neurons and the extent of mC4 overexpression in transfected cells at P21.

IUEs reliably increased expression of mC4 at P21 in excitatory CaMKIIα+ neurons of the mPFC relative to their nearby untransfected neighbors (S1D Fig, *t* test, ****p < 0.0001). Furthermore, we found that 99% of cells expressing GFP had higher amounts of mC4 mRNA relative to the mean transcript levels of nearby untransfected cells (S1E Fig). To quantify mRNA levels more precisely, we performed quantitative PCR (qPCR) in tissue that was dissected to isolate the transfected region of the mPFC (Fig 1B, right panel) from control (IUE with pCAG-GFP) and mC4 (IUE with pCAG-GFP and pCAG-mC4) animals at P21 and P60. At P21, IUEs reliably increased mC4 transcript by approximately 2.8-fold relative to control (Fig 1C). However, there was not a significant increase of mC4 mRNA at P60 in the mC4 condition (Fig 1D).

We tested multiple commercial antibodies in order to detect mC4 protein. However, they were either nonspecific and/or not sensitive enough to detect C4 upon transient transfection of human epithelial kidney (HEK) cells. Therefore, we generated an antibody that detected both mC4 and hC4 in HEK cells that were transfected with C4 (S2A Fig). Using this antibody, we found that C4 protein expression was increased in the mC4 condition approximately 1.3-fold at P21 and was unchanged at P60 in pooled total cell lysates taken from the mPFC (Fig 1E). To test whether C4 is synaptically localized, we isolated postsynaptic density (PSD) fractions from PFC tissue (S2B and S2C Fig). Indeed, we found that C4 protein was present in the PSD fraction in control conditions and synaptic C4 protein was increased approximately 1.6-fold at P21 and was unchanged at P60 in C4-overexpressing tissue compared to controls (Fig 1E). Since we had to pool isolated PSDs from multiple mice to have enough synaptic material, we were unable to run statistics on this data.

We were unable to detect either endogenous or exogenous C4 in histological sections using our antibody or other commercially available antibodies. Therefore, we created a C4-GFP fusion protein construct (pCAG-C4-GFP) to visualize exogenous C4 protein expression without the need for antibodies. We confirmed C4-GFP expression in HEK cells using western blot (S2A Fig). In both transfected HEK cells and L2/3 neurons at P21, mC4-GFP protein was expressed (S3A and S3B Fig). We found C4-GFP localized to cell bodies and dendrites in L2/3 neurons (S3B Fig, yellow arrowhead). Overall, these results showed that mPFC L2/3 neurons express endogenous mC4, that a subpopulation of C4 is synaptically localized, and that IUE successfully increased mC4 levels at P21.

### C4 overexpression causes dendritic spine alterations in apical arbors of L2/3 neurons

Since we found C4 in PSD fractions, we wondered whether increased expression of C4 in developing neurons led to structural alterations in dendritic spines. We used IUE to produce control (pCAG-GFP) or C4 overexpression conditions (pCAG-GFP with pCAG-mC4b or pCAG-hC4A, for mC4 and hC4 conditions, respectively). Since early postnatal development is an important period for synaptic maturation and refinement of cortical circuitry [38], we measured dendritic spine density using confocal imaging of GFP in fixed brain sections collected at P7–9, P14–16, P21–23, and P55–60. Besides control and mC4 conditions, for comparison we also electroporated hC4A, which is highly associated with SCZ [16].

**Fig 2:**
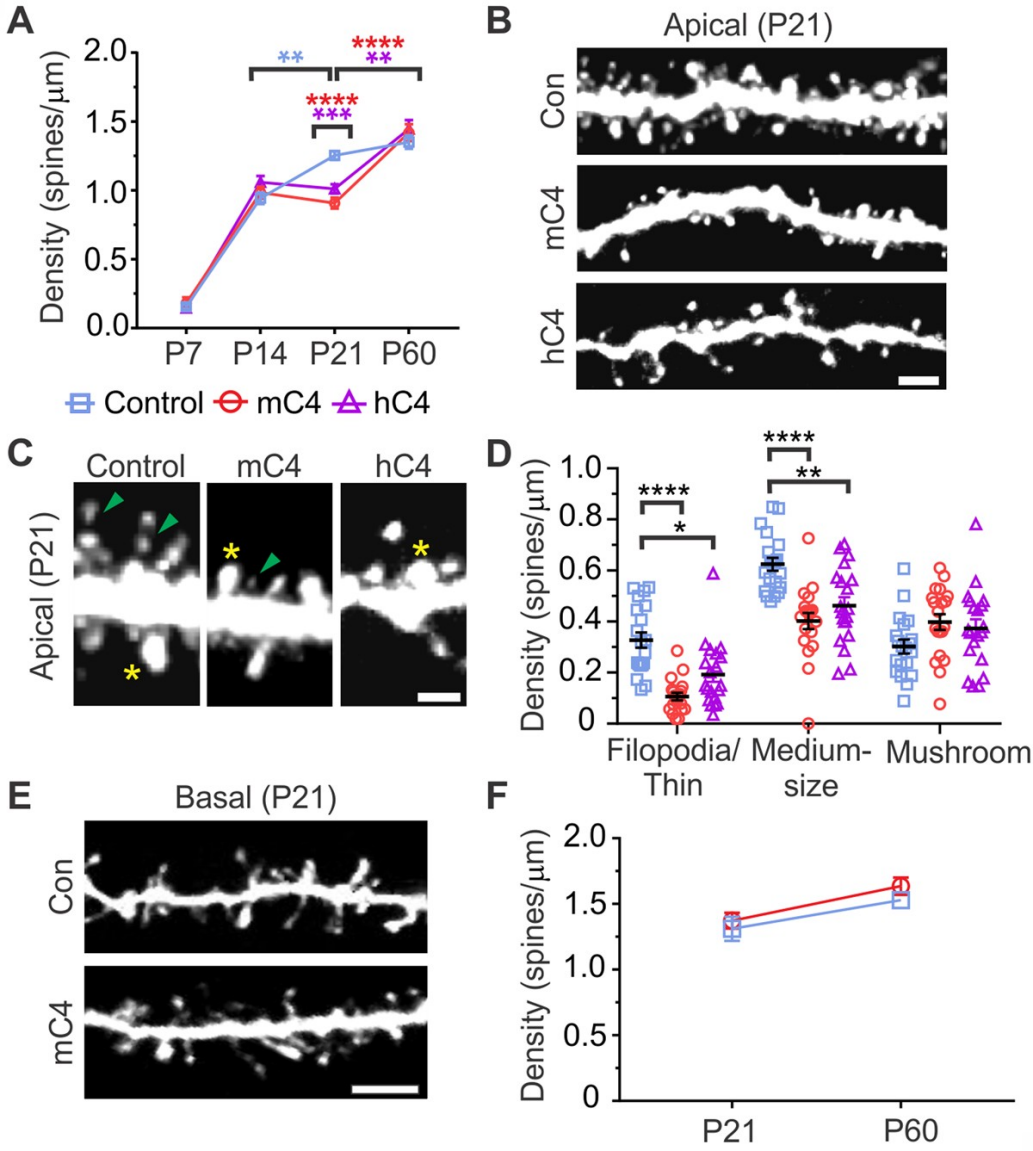
C4 overexpression led to dendritic spine alterations in apical arbors of L2/3 mPFC neurons. (A) Developmental time course of spine density in the mPFC revealed a significant decrease in spine density (spines/μm) in neurons overexpressing C4, as compared to controls (“Con”), at P21–23. **p < 0.01, ***p < 0.001, ****p < 0.0001. *N* = 240 dendrites from 84 mice (20 dendrites per each time point; 20 dendrites × 4 time points × 3 conditions). (B) Representative 40X confocal images of P21–23 apical dendritic tufts. Scale bar = 5 μm. (C) Representative 40X confocal images of P21–23 apical dendritic spine types. Yellow asterisk: large mushroom spine (TIB [a.u.] > 75%). Green arrowhead: thin spine/filopodia (TIB [a.u.] < 25%). Scale bar = 3 μm. (D) Spine density (spines/μm) sorted by spine types reveals a specific reduction of medium-sized and thin/filopodia spine types in the mC4 and hC4 condition. *p < 0.05, **p < 0.01, ****p < 0.0001. *N* = 60 dendrites from 21 mice (20 dendrites per condition × 3 conditions). (E) Representative 40X confocal images of P21–23 basal dendritic spines. Scale bar = 5 μm. (F) Analysis of basal dendritic spine density (spines/μm) revealed no difference across groups. *N* = 80 dendrites from 28 mice (20 dendrites per each time point; 20 dendrites × 2 time points × 2 conditions). Two-way ANOVA followed by a Tukey’s test for all comparisons. Control: blue; mC4: red; hC4: purple. Mean ± SEM. For underlying data, see https://osf.io/7em3s/?view_only=0e7ffde4ebd344dc83af83b5a605c451. a.u., arbitrary units; hC4, human C4; L2/3, layer 2/3; mC4, mouse C4; mPFC, medial prefrontal cortex; P, postnatal day; TIB, total integrated brightness.

In control conditions, the density of apical tuft dendritic spines was developmentally regulated and increased nearly 8-fold in the 2 wk from P7 to P21 (Fig 2A, Tukey’s test, ****p < 0.0001). Whereas spine density in mC4- or hC4-overexpressing neurons was similar to that of control conditions at P7 and P14, apical tuft dendritic spine density was approximately 30% lower at P21 (Fig 2A and 2B). There were no differences in apical tuft protrusion density between any groups at P60 (Fig 2A). These results suggest that overexpression of C4 alters the developmental maturation of dendritic spines and that overexpression of either human or mouse homologues of C4 causes spine loss/dysgenesis in the mouse mPFC.

Closer inspection of protrusion morphology at P21 revealed a significant reduction of thin spines/filopodia and medium-sized spines in neurons overexpressing either mC4 or hC4, as compared to controls, whereas putative large mushroom spines remained intact (Fig 2C and 2D; spine types sorted by total integrated brightness [TIB], see Methods). Since dendritic spine volume positively correlates with PSD size and synaptic strength [39,40], this result suggests that increased expression of mC4 preferentially alters the development of weaker synaptic connections. Since overexpression of mC4 and hC4 led to similar defects in dendritic spine density at P21, in subsequent experiments we focused on the mouse homologue to investigate the role of C4 in cortical circuit function. In contrast to the structural alterations we observed in apical tufts, increased expression of mC4 did not significantly alter the density of basal dendritic spines, as compared to control conditions (Fig 2E and 2F). Taken together, these results demonstrate that increased expression of C4 in developing cortical neurons caused input-specific dendritic spine pathology and that less mature protrusions were preferentially affected, whereas larger mushroom spines remained intact.

### C4 overexpression reduced functional connectivity in cortical neurons

**Fig 3:**
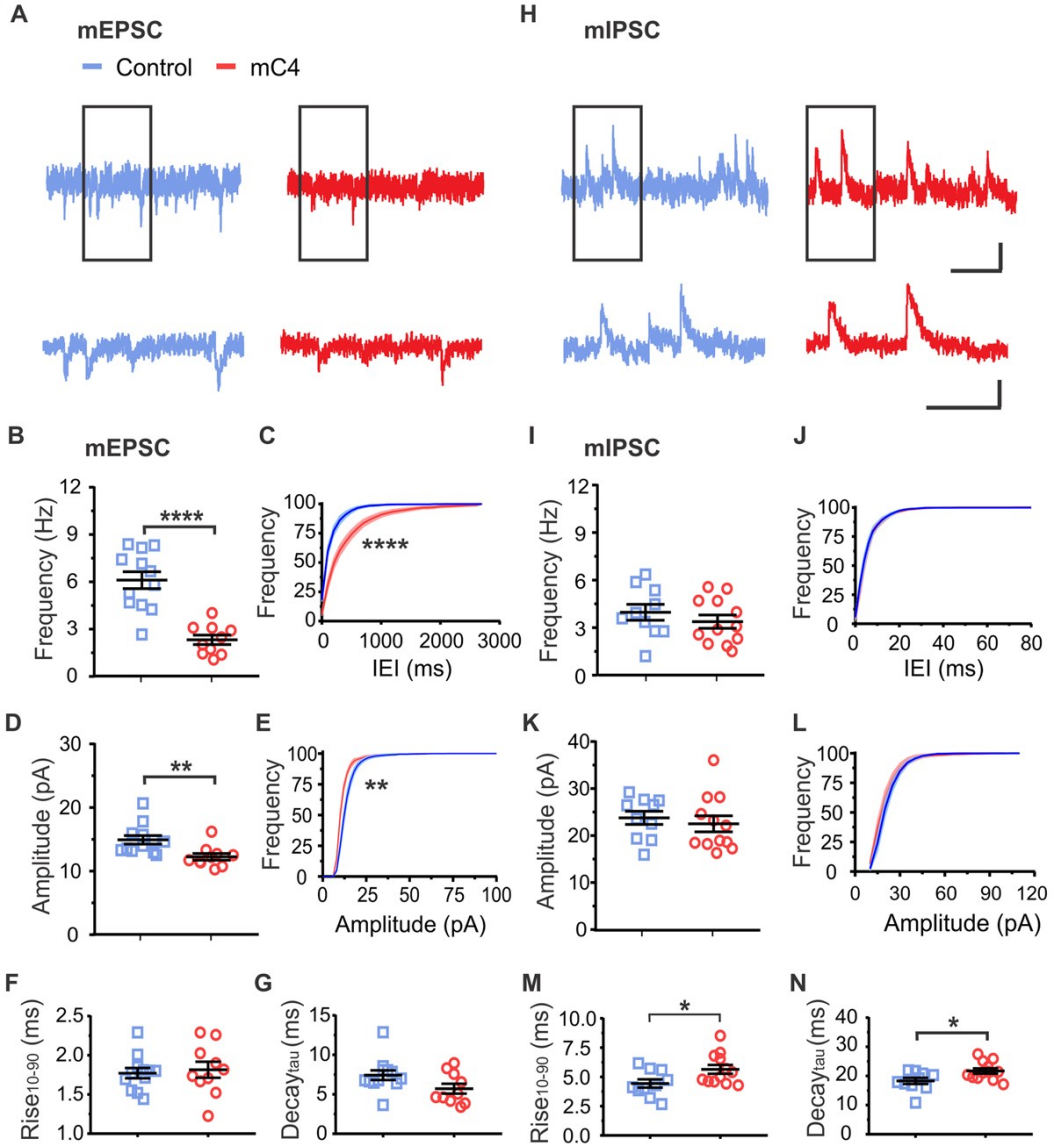
C4 overexpression reduced functional connectivity in cortical neurons. (A) Top: Representative whole-cell voltage-clamp recordings showing mEPSCs. Top scale bar = 250 ms/10 pA. Bottom: Same as top traces but expanded (black rectangle region). Bottom scale bar = 125 ms/10 pA. (B) Increased mC4 expression caused a reduction in mEPSC frequency. *t* test. ****p < 0.0001. (C) Overexpression of mC4 caused a rightward shift in the distribution of mEPSC IEI. Kolmogorov-Smirnov test. ****p < 0.0001. (D) mC4 caused a reduction in mEPSC amplitude. *t* test. **p < 0.01. (E) mC4 overexpression caused a leftward shift in the mEPSC amplitude distribution. Kolmogorov-Smirnov test. **p < 0.01. (F) mEPSC Rise_10–90_ was not altered by mC4 overexpression. *t* test. *p* = 0.715. (G) No changes in mEPSC Decay_tau_ with mC4 overexpression. *t* test. *p* = 0.07. (B-G) *N* = 12 control neurons, *N* = 10 mC4 neurons. Mean ± SEM. Control: blue; mC4: red. (H) Top: Representative recordings showing mIPSCs. Top scale bar = 250 ms/20 pA. Bottom traces are same as (H) top but expanded (rectangle region). Top scale bar = 125 ms/20 pA. (I) No difference in mIPSC frequency between control and mC4 conditions. *t* test. *p* = 0.3726. (J) Distribution of mIPSC IEIs was not changed by increased expression of mC4. Kolmogorov-Smirnov test. *p* > 0.05. (K) No changes in mIPSC amplitude with increased expression of mC4. *t* test. *p* = 0.5832. (L) mIPSC amplitude distribution was not changed by increased expression of mC4. Kolmogorov-Smirnov test. *p* > 0.05. (M) mIPSC Rise_10–90_ was increased in neurons overexpressing mC4. *t* test. *p = 0.0329. (N) mIPSC Decay_tau_ was increased in neurons overexpressing mC4. *t* test. *p = 0.0225. (I-N) *N* = 10 control neurons, *N* = 12 mC4 neurons. Control: blue; mC4: red. Mean ± SEM. For underlying data, see https://osf.io/7em3s/?view_only=0e7ffde4ebd344dc83af83b5a605c451. IEI, interevent interval; mC4, mouse C4; mEPSC, miniature excitatory postsynaptic current; mIPSC, miniature inhibitory postsynaptic current.

To determine whether the morphological spine deficits observed in C4-overexpressing cells were accompanied by functional connectivity changes, we performed electrophysiological whole-cell voltage-clamp recordings in acute brain slices prepared from mPFC of P18–25 control and mC4 animals. Voltage-clamp recordings demonstrated that overexpression of mC4 caused an approximately 60% decrease in the frequency of miniature excitatory postsynaptic currents (mEPSCs) and an increase in the interevent interval (IEI) (Fig 3A–3C). Increased levels of mC4 also caused an approximately 18% reduction in the amplitude of mEPSCs (Fig 3D and 3E), demonstrating that mC4 modifies not only the density of synaptic connections but also the postsynaptic responses of L2/3 neurons.

Further analysis of postsynaptic current kinetics revealed no differences in mEPSC Rise_10-90_ or Decay_tau_ between control and mC4-transfected neurons (Fig 3F and 3G). Although overexpression of mC4 did not alter the frequency or the amplitude of miniature inhibitory postsynaptic currents (mIPSCs) (Fig 3H–3L), it significantly slowed their kinetics (Fig 3M and 3N, 27% and 19% increase in Rise_10–90_ and Decay_tau_, respectively), suggesting that there could be mC4-dependent changes in the location of inhibitory synapses. Taken together, our data suggest that increased expression of mC4 alters the developmental wiring of cortical neurons by decreasing their excitatory synaptic drive.

### Overexpression of mC4 decreased membrane capacitance without altering overall excitability

**Fig 4:**
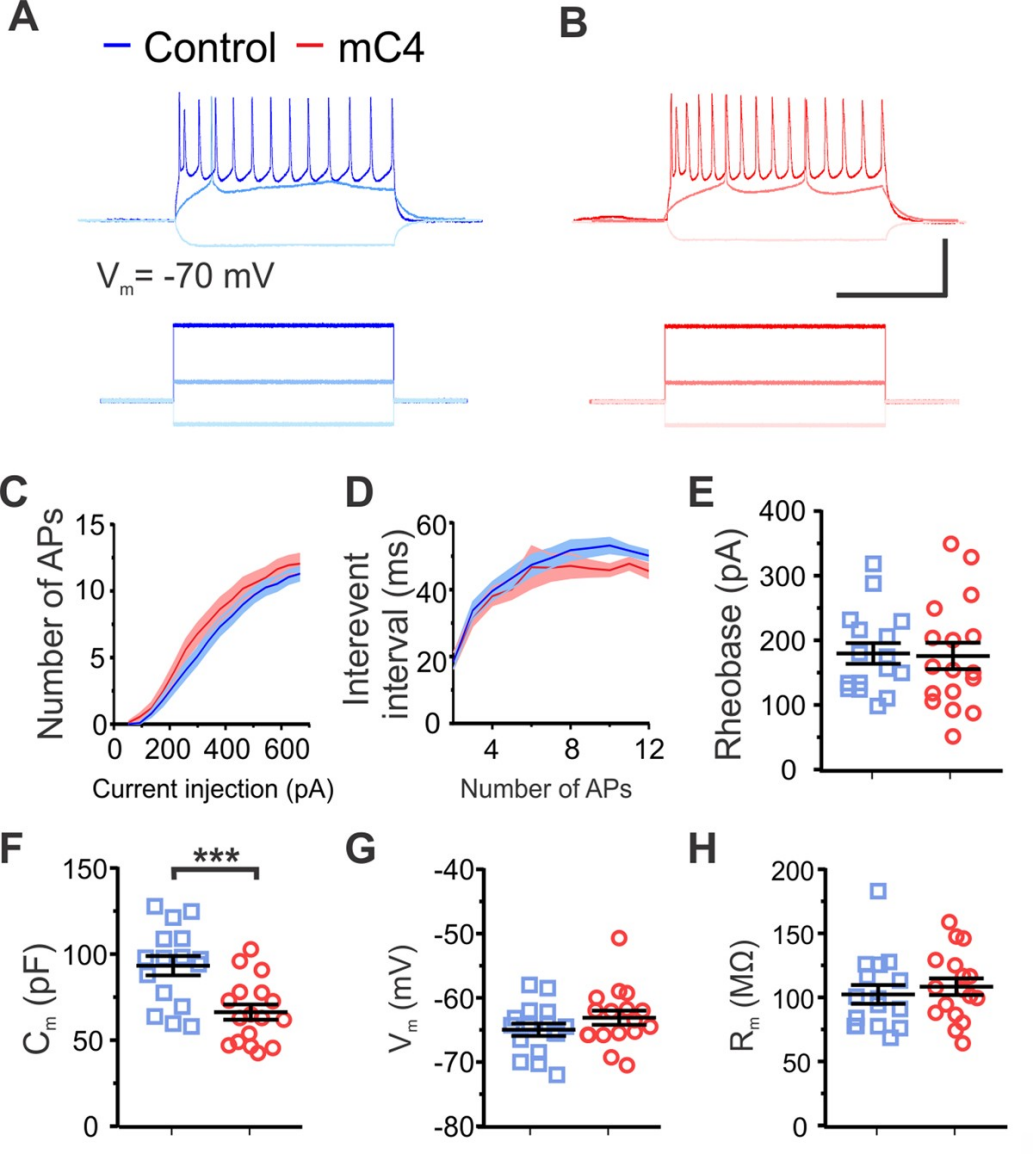
Overexpression of mC4 decreased membrane capacitance without altering overall excitability. (A-B) Representative current-clamp recordings from control (A) and mC4 (B) neurons in response to injection of constant-current pulses. Voltage traces (top) shown for response to −220 pA (light blue/pink), 185 pA (medium blue/red), and 685 pA (dark blue/red) current injections (bottom). Scale bar = 500 pA or 50 mV. Scale bar = 250 ms. (C) The number of APs was not different between conditions. Dark blue trace: control mean. Light blue trace: control SEM. Dark red trace: mC4 mean. Light red trace: mC4 SEM. Two-way ANOVA. *p* = 0.9989. (D) Interevent interval was not different between conditions. Dark blue trace: control mean. Light blue trace: control SEM. Dark red trace: mC4 mean. Light red trace: mC4 SEM. Two-way ANOVA. *p* = 0.9838. (E) Rheobase was not altered by the overexpression of mC4. *t* test. *p* = 0.8795. (F) mC4 overexpression led to a dramatic reduction in C_m_. *t* test. ***p = 0.0007. (G) V_m_ was not changed by mC4 overexpression. *t* test. *p* = 0.2166. (H) R_m_ was not affected by mC4 overexpression. *t* test. *p* = 0.5455. (C-H) Control: *N* = 16 cells; mC4: *N* = 17 cells. Blue: control. Red: mC4. Mean ± SEM. For underlying data, see https://osf.io/7em3s/?view_only=0e7ffde4ebd344dc83af83b5a605c451. AP, action potential; mC4, mouse C4.

We also monitored the electrophysiological properties of L2/3 mPFC neurons to determine whether overexpression of mC4 altered neuronal excitability. To do this, we injected hyperpolarizing and depolarizing current step injections of different amplitudes and recorded the membrane potential (V_m_) in control and mC4-overexpressing neurons (Fig 4A and 4B). Overexpression of mC4 did not alter the number of action potentials (APs) in response to step current injections of incrementally increasing amplitudes (Fig 4C). In agreement with this, we did not see a change in the IEI of APs in response to step current injections (Fig 4D) or the minimal current injection that resulted in AP generation (Fig 4E) in mC4-overexpressing neurons. Together, these results suggest that overexpression of mC4 did not alter the intrinsic excitability of cortical neurons.

In contrast, increased levels of mC4 led to an approximately 29% decrease in the membrane capacitance relative to controls (Fig 4F). This effect on passive membrane properties was specific because increased expression of mC4 did not alter the neuron’s resting membrane potential or membrane input resistance (Fig 4G and 4H). Since membrane capacitance is proportional to a cell’s surface area, this result suggests that increased expression of mC4 could affect either the gross morphology of cortical neurons or plasma membrane properties. Further investigation of neuronal morphology showed no difference between conditions in cell body area/diameter or in the width of primary dendrites (S4 Fig); there was also no change in dendritic complexity (S5 Fig), suggesting that gross neuronal morphology was not affected by mC4 overexpression. Besides the mC4-dependent changes in membrane capacitance, increased expression of mC4 did not have an effect on the morphology or intrinsic properties of transfected cells, thus suggesting that overall neuronal health was not affected by increased expression of mC4.

### mC4 overexpression enhanced microglia engulfment of postsynaptic PSD-95

Several lines of evidence suggest that microglia contribute to circuit maturation and synaptic refinement through the secretion of effector molecules and direct interactions with neurons [41]. To determine whether increased expression of mC4 led to closer associations of microglia with neurons at P21, we measured the colocalization of transfected neuronal GFP with microglia (immunostained for ionized calcium binding adaptor molecule 1 [Iba1]) in single z-planes using confocal microscopy (S6A Fig). Using this approach, we found instances of microglia colocalized with GFP+ neuronal processes in both conditions (S6B Fig; white arrowheads), suggesting that microglia were in close proximity with the dendritic processes of transfected L2/3 neurons. Increased expression of mC4 led to an approximately 35% increase in the number of microglia that colocalized with GFP+ L2/3 neurons (S6C Fig) and an approximately 2-fold increase in the area of GFP signal that colocalized with the microglia cell body and proximal processes (S6D Fig). These results suggest that neuronal overexpression of mC4 led to an increase in the number of microglia in close proximity to neurons despite no change in the overall density of microglia in the transfected region (S7 Fig).

Since increased expression of mC4 led to a decrease in synaptic connectivity (Figs 2 and 3) and closer association of microglia with transfected neurons at P21 (S6 Fig), we evaluated whether C4 overexpression also enhanced microglia-dependent phagocytosis of synaptic material. To do this, endogenous PSD-95 was fluorescently labeled in vivo using a plasmid containing PSD95-FingR (EF1a-PSD95.FingR-RFP) [42], which labeled only endogenous PSD-95 within transfected neurons with red fluorescent protein (RFP) (S8 Fig; pseudocolored green). Fluorescent signal from PSD95-FingR was confined to superficial L1 and L2/3 including transfected cell bodies of L2/3 neurons, indicating that expression from this electroporated construct mirrored the expected PSD-95 expression pattern of L2/3 neurons [43,44] (S8 Fig).

**Fig 5:**
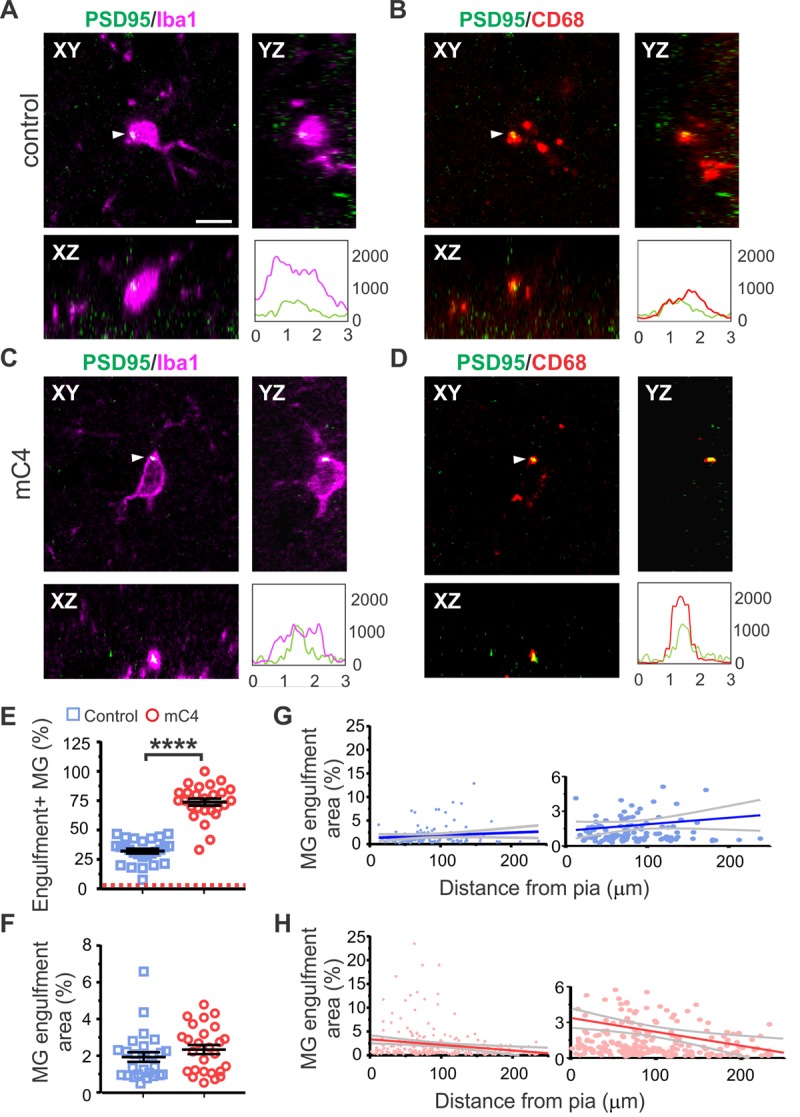
mC4 overexpression enhanced microglia engulfment of postsynaptic PSD-95. (A-D) Representative confocal image (60X) showing PSD-95 located within microglial lysosomes in P21 mice for control (A-B) and mC4 conditions (C-D). Single z-plane shown. Scale bar = 5 μm. Magenta: microglia (“MG) (Iba1). Red: lysosomes (CD68). Green: PSD-95 (PSD95-FingR-RFP, pseudocolored green). White arrowheads indicate colocalization of PSD95 with Iba1 (A and C) or CD68 (B and D). Panels A and C show a representative microglia (for control and mC4, respectively) including Iba1 and PSD95-FingR-RFP signal. Panels B and D show the same z-plane as panels A and C but shows CD68 and PSD95-FingR-RFP signal. Orthogonal views shown. Panels A-D each include a graph (bottom right panel) showing a line intensity scan for each signal (panels A and C show Iba1 [magenta] and PSD-95 (green); panels B and C show CD68 [red] and PSD-95 [green]). For line intensity graphs, y-axis shows gray intensity value (a.u.), and x-axis shows length (μm). (E) mC4 overexpression increased the number of microglia positive for PSD-95 engulfment (PSD95-FingR-RFP signal colocalized with CD68 and Iba1). *t* test. ****p < 0.0001. Red dotted line: average of control and C4 in shuffled pixel analysis. (F) There was no difference in area of PSD95 colocalized with microglia lysosomes between conditions. (MG engulfment area % = area of microglia occupied by PSD95-FingR signal in lysosomes / total microglia area). *t* test. *p* = 0.1927. (E-F) Data points represent averages from transfected region ROIs. Control: *N* = 26 ROIs (from 5 mice; 345 microglia). mC4: *N* = 26 ROIs (from 5 mice; 319 microglia). Mean ± SEM. (G-H) Microglia engulfment area (%) (from panel F) as a function of cortical depth (μm) for control (G) and mC4 (H). Data points represent individual microglia. *N* = 345 control and *N* = 319 mC4 microglia. Right graphs are same as left but are zoomed on the y-axis. Blue line: control mean. Red line: mC4 mean. Gray lines: 95% confidence intervals. Pearson’s r correlation. Control: r = 0.12. *p* > 0.05. mC4: r = −0.21. **p < 0.01. For underlying data, see https://osf.io/7em3s/?view_only=0e7ffde4ebd344dc83af83b5a605c451. a.u., arbitrary units; Iba1, ionized calcium binding adaptor molecule 1; mC4, mouse C4; P, postnatal day; PSD, postsynaptic density; ROI, region of interest.

Next, we quantified the colocalization of PSD-95 (identified by PSD95-FingR immunofluorescence) with microglia (Iba1) or microglial lysosomes (CD68) (Fig 5). We observed clear instances of PSD-95 colocalized with Iba1 (Fig 5A and 5C, white arrowheads) and CD68 signal (Fig 5B and 5D, white arrowheads), suggesting that synaptic material was engulfed by microglia and localized to microglia lysosomes. Furthermore, using orthogonal views, we confirmed that PSD-95 was localized within microglia soma and lysosomal compartments (Fig 5A–5D). We quantified the colocalization of fluorescent signals from PSD-95-FingR with both Iba1 and CD68 to measure postsynaptic puncta within the lysosomes of microglia in control and mC4 conditions. Overexpression of mC4 led to an approximately 2.3-fold increase in the percentage of microglia positive for engulfment (Fig 5E). As a control, we analyzed the same images after pixel shifting (see Methods). In the pixel-shifted data, the percentage of microglia positive for triple-stained puncta dropped to under 3% in both groups (Fig 5E, red dotted line is mean shifted value for both groups), suggesting that the colocalization of signals in microglia did not occur by chance. Lastly, we observed no change in the area of PSD-95-FingR signal within CD68+ structures (Fig 5F). Taken together, these data show that C4 overexpression drives microglia engulfment of synaptic material.

Since increased expression of mC4 led to specific spine loss in L1 apical tufts with no changes in basal dendrites (Fig 2), we tested whether microglia engulfment of synaptic material was more prominent near the apical dendrites of cortical neurons. We measured the distance from the center of each microglia soma to the pia to identify its cortical depth, and this measurement was then compared to the area of microglia-engulfed PSD-95 signal. This allowed us to determine whether the connectivity deficits in apical tufts were due to altered microglia phagocytosis of synapses in a layer-specific manner. In control conditions, there was no correlation between the amount of PSD-95 colocalized with microglia and cortical depth, suggesting that, during normal development, microglia were not biased toward phagocytosis of PSD-95 in specific layers (Fig 5G). However, we observed a significant correlation in the mC4 condition such that microglia closer to L1, where apical tufts are located, engulfed more PSD-95 material than their counterparts in deeper layers (Fig 5H). To test whether mC4-dependent enhancement of phagocytosis in L1 was due to an increased density of microglia, we quantified microglia density in L1 (cortical depth: 0–120 μm) and L2/3 (cortical depth: >120–300 μm) and found no difference between microglia density between layers in either condition (S7 Fig). There was also no difference in CD68 area within microglia between control and mC4 conditions (S7 Fig). In summary, our data support the hypothesis that C4 overexpression drives layer-specific circuit dysfunction through excessive microglia engulfment of postsynaptic material.

Additionally, we used expansion microscopy (ExM), a biological sample–preparation procedure that allows for nanoscale imaging with standard confocal microscopes [45] (S9 Fig), to confirm colocalization of fluorescent signals from PSD-95 and CD68 located in microglia. Using ExM, we increased the size of microglia soma by approximately 180% (equivalent to approximately 2.8× linear expansion) in control and mC4 conditions at P21 (S9D–S9F Fig). Microglia cell body size increased similarly along its long and short axis, supporting previous findings that have shown that ExM expands biological tissue linearly [45] (S9D–S9F Fig). Other than increasing their size, expanded microglia had morphologies similar to cells in pre-expansion conditions (S9 Fig), suggesting that ExM did not fundamentally alter the structure of these brain-resident macrophages.

**Fig 6:**
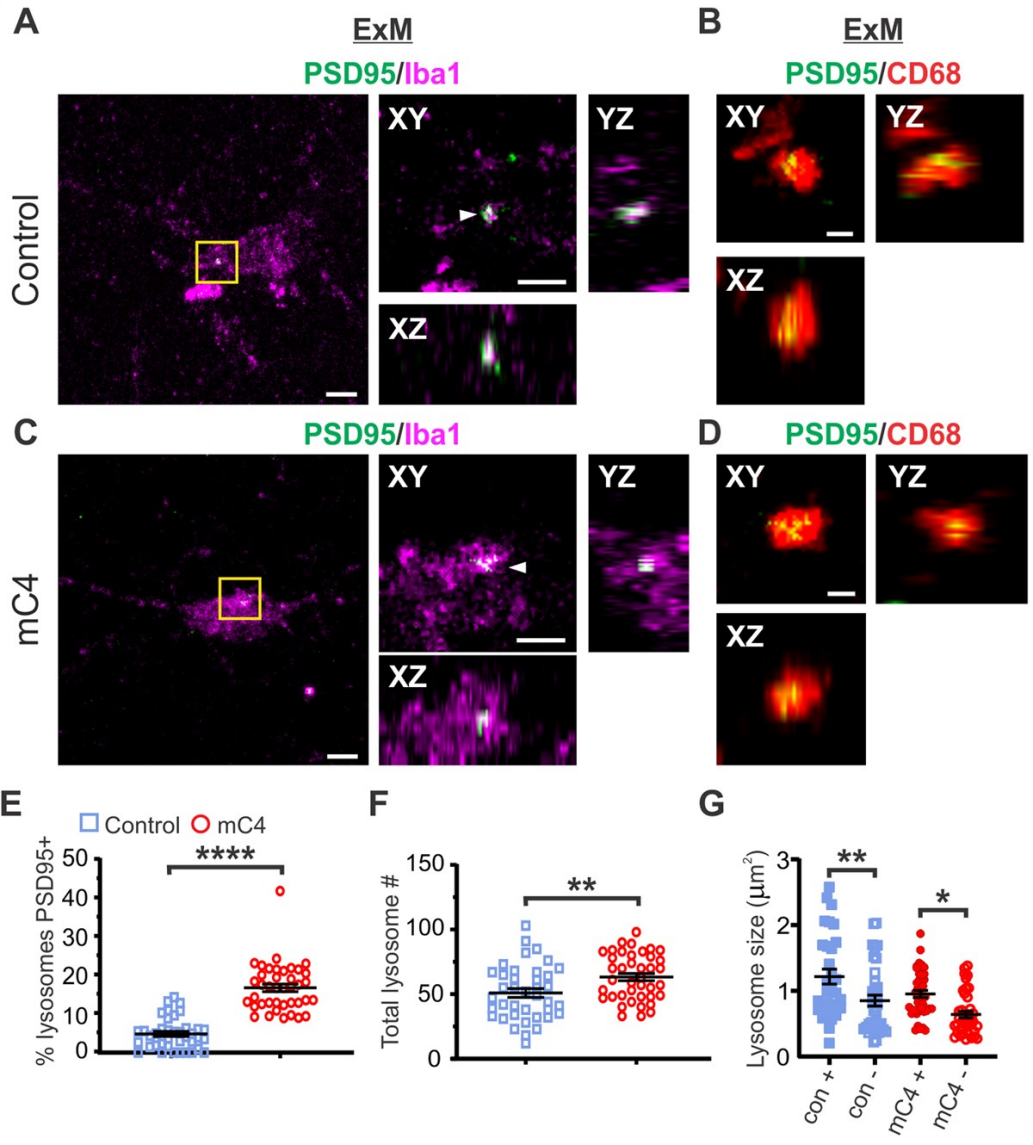
ExM confirmed C4 overexpression led to enhanced microglial engulfment of PSD-95. (A-D) Representative confocal images (40X) in expanded tissue showing PSD-95 within microglial lysosomes in P21 mice for control (“con”) (A and B) and mC4 (C and D) conditions. Panels A and C show a representative microglia (for control and mC4, respectively) including Iba1 and PSD95-FingR-RFP (pseudocolored green) signal. White arrowheads indicate colocalization of PSD-95 with Iba1 (A and C). Panels B and D show the same z-plane as panels A and C but with CD68 and PSD95-FingR-RFP signal. Orthogonal views shown (XY, YZ, and XZ). All images are single z-planes. Magenta: microglia (Iba1). Red: lysosomes (CD68). Green: PSD-95 (PSD95-FingR-tagRFP, pseudocolored green). Yellow box in (A and C) shows zoomed region for orthogonal views. (A and C) Left scale bar = 5 μm, right scale bar (in XY plane) = 2.5 μm. (B and D) Scale bar = 1 μm. (E) mC4 overexpression led to an increase of lysosomes that were positive for PSD-95 signal. The percent of lysosomes that were PSD95 positive per each microglia compared between control and mC4. *t* test. ****p < 0.0001. (F) Number of lysosomes in microglia was increased in the mC4 condition relative to controls. *t* test. **p = 0.0064. (G) Lysosomes that were positive for PSD-95 were larger in size compared to PSD-95(−) lysosomes in both control (“con”) and mC4 conditions. Lysosome size (μm^2^) per microglia for control and mC4 conditions separated into lysosomes positive or negative for PSD-95 signal. Control (+) and mC4 (+) are lysosome size for lysosomes positive for PSD95. Control (−) and mC4 (−) are lysosome size for lysosomes that are negative for PSD95. Two-way ANOVA followed by a Tukey’s test for all comparisons. Control (+ versus −): **p = 0.0071. mC4 (+ versus −): *p = 0.0150. Control (+) versus mC4 (+): *p* = 0.0868. Control (−) versus mC4 (−): *p* = 0.2987. (E-G) Blue: control. Red: mC4. *N* = 86 microglia (45 control [from 5 mice] and 41 mC4 [from 4 mice] microglia). Mean ± SEM. For underlying data, see https://osf.io/7em3s/?view_only=0e7ffde4ebd344dc83af83b5a605c451. ExM, expansion microscopy; Iba1, ionized calcium binding adaptor molecule 1; mC4, mouse C4; P, postnatal day; PSD, postsynaptic density.

Using ExM, we observed clear examples of PSD-95 colocalized with Iba1 (Fig 6A and 6C, white arrowheads) and CD68 signal (Fig 6B and 6D), suggesting synaptic material was localized to microglia lysosomes. Confocal images obtained after ExM show examples of microglia lysosomes that were both negative and positive for PSD-95 in control and mC4 conditions (S10 Fig). Overexpression of mC4 in L2/3 neurons led to an approximately 3.3-fold increase in lysosomes positive for PSD-95 (Fig 6E). Additionally, microglia in the mC4 condition contained significantly more lysosomes relative to control (Fig 6F, approximately 1.2-fold increase). In contrast, previously we found no change in CD68 reactivity using conventional confocal microscopy (S7B Fig); this could be due to the enhanced resolution made possible by ExM. For both conditions, lysosomes positive for PSD-95 were approximately 1.5-fold larger than those that were negative for postsynaptic material (Fig 6G). Additionally, lysosome size did not differ between conditions for PSD-95(+) lysosomes (con[+] versus mC4[+]) or for PSD-95(−) lysosomes (con[−] vs mC4[−]). This suggests that lysosomes positive for PSD-95 were morphologically different than lysosomes without synaptic material for both control and mC4 conditions. In summary, our data support the hypothesis that C4 overexpression drives layer-specific circuit dysfunction through excessive microglia engulfment of postsynaptic material.

### Complement-dependent alterations in frontal cortical circuitry were sufficient to alter social interactions in juvenile and adult mice

To determine whether overexpression of mC4 in the frontal cortex is sufficient to cause deficits in early (P18) social behaviors, we administered a task that allowed us to measure sensorimotor abilities of mice and maternal–pup social interactions [46]. For these experiments, we used a modified IUE method (see Methods) to target large populations of L2/3 neurons in both hemispheres of the frontal cortex. Post hoc analysis of brains from these mice showed that most transfected cells were in prefrontal cortical regions, confirming that we were able to increase mC4 expression in large populations of PFC L2/3 neurons (S11 Fig).

**Fig 7:**
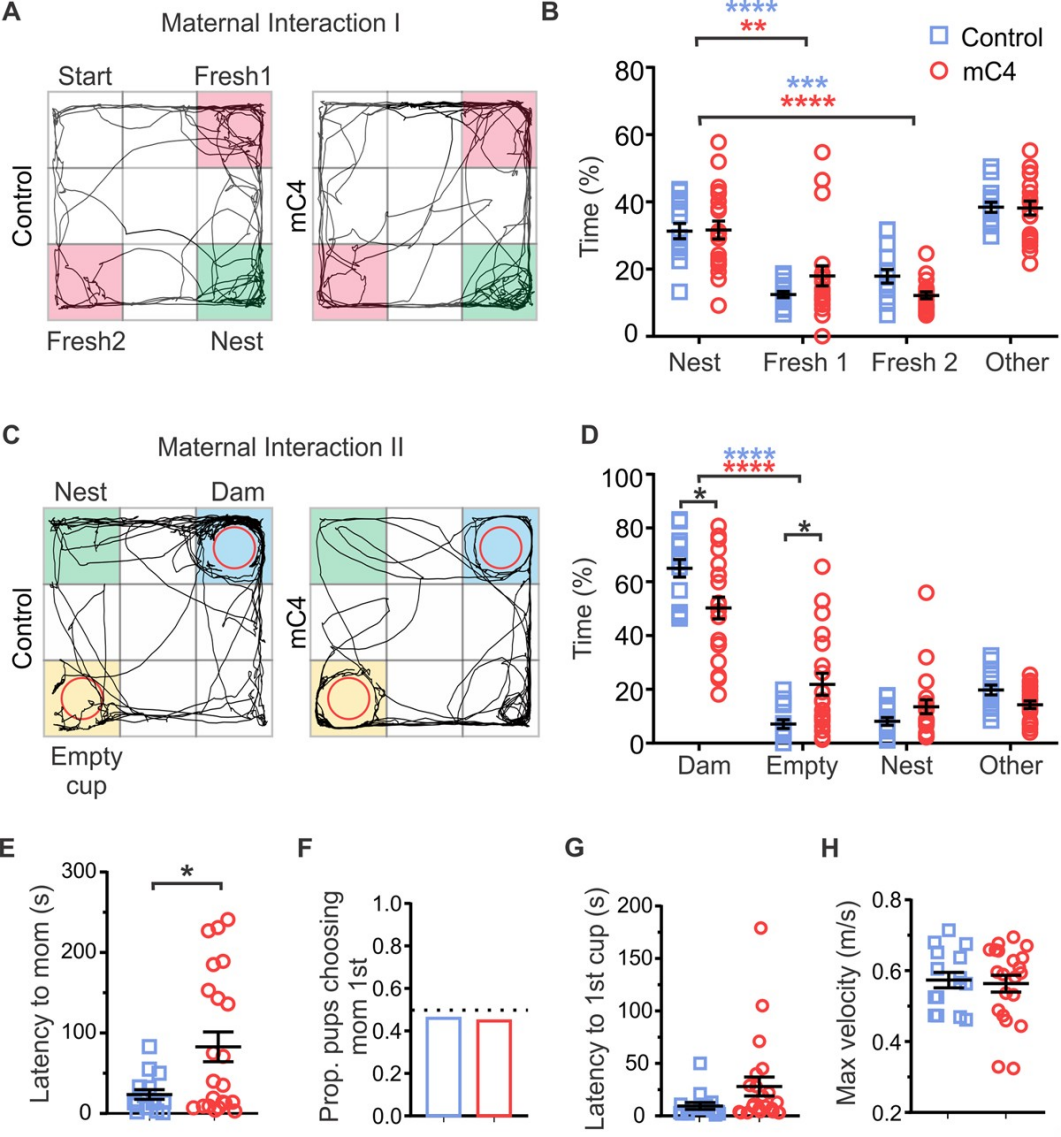
C4-dependent alterations in frontal cortical circuitry were sufficient to alter maternal–pup social interactions. (A) Representative examples of path traveled (black trace) by P18 control and mC4 pups in MI1 task. Fresh bedding corners (Fresh 1 and 2, pink) and nest bedding corner (green). (B) Control and mC4 pups spent a similar proportion of time exploring the nest and fresh corners in the MI1 task, suggesting motor and sensory skills were intact. All mice spent more time in nest bedding than in fresh bedding. **p < 0.01, ***p < 0.001, ****p < 0.0001. Two-way ANOVA and Sidak's posttest. Control versus mC4 time spent in nest: *p* > 0.9999. Control versus mC4 time spent in Fresh 1: *p* = 0.3327. Control versus mC4 time spent in Fresh 2: *p* = 0.3138. (C) Representative examples of path traveled (black trace) by P18 control and mC4 pups in MI2 task. Dam’s cup (dam: blue), empty cup (empty cup: yellow), nest bedding corner (nest: green). (D) mC4 pups spent less time interacting with the dam and more time near the empty cup compared to controls. Two-way ANOVA and Sidak's posttest. *p < 0.05. ****p < 0.0001. (E) mC4 mice took longer to approach the dam relative to controls (s). *t* test with Welch's correction. *p < 0.05. (F) Control and mC4 pups traveled to dam’s cup first about 50% of the time, suggesting first choice was random. Fisher’s exact test. *p* = 0.9999. (G) Latency to reach first cup (either dam or empty) was not different. *t* test with Welch's correction. *p* = 0.06. (H) Control and mC4 pups reached the same maximum velocity (m/s). *t* test. *p* = 0.77. (B, D-H) *N* = 15 control mice and *N* = 21 mC4 mice. Blue: control. Red: mC4. Mean ± SEM unless otherwise noted. For underlying data and tracking script, see https://osf.io/7em3s/?view_only=0e7ffde4ebd344dc83af83b5a605c451. mC4, mouse C4; MI1, maternal interaction 1; MI2, maternal interaction 2; P, postnatal day; Prop, proportion.

To test sensorimotor abilities (maternal interaction 1 [MI1] task), we transferred P18 pups to an arena that contained fresh bedding in two neutral corners and nesting material from the animal’s home cage in the corner opposite to the starting corner, and mice were free to explore the arena for 3 min (Fig 7A). Similar to controls, mC4 mice spent more time exploring the nest corner relative to the neutral corners, suggesting that mC4 mice had normal homing behavior (Fig 7B). Although grooming occurrences of mC4 mice were more frequent and about 4.3-fold longer (S12 Fig) than in control mice, there was no interaction between experimental group and corner preference (Fig 7B). These results indicate that although mC4 mice engaged in more repetitive behavior, they were able to explore the arena and interact with their nest bedding to the same extent as control animals. Additionally, these results suggest that increased levels of mC4 in the frontal cortex did not impair overall sensory abilities or cause gross motor deficits in P18 pups.

To test maternal–pup social interactions (maternal interaction 2 [MI2] task), we placed P18 pups in an arena that contained nest bedding, an empty wire mesh cup, and a wire mesh cup containing the pup’s dam, and the behavior of each pup was monitored for 5 min (Fig 7C). We found that mC4 mice showed an approximately 23% reduction in time spent exploring the cup containing the dam as compared to control mice (Fig 7D). mC4 mice also exhibited an approximately 2.5-fold decrease in the latency to first approach the dam’s cup (Fig 7E). The proportion of pups that approached the dam’s cup before the empty cup (at start of trial) was approximately 50% (Fig 7F), suggesting that pups in both conditions engaged in similar exploratory behavior of the arena before seeking their mother. mC4 and control mice had similar latencies to first reach either the empty or dam’s cup (Fig 7G) and similar maximum speeds (Fig 7H). Taken together, these results indicate that mC4 pups had intact motor and sensory abilities but were less motivated or interested in interacting with their mother.

**Fig 8:**
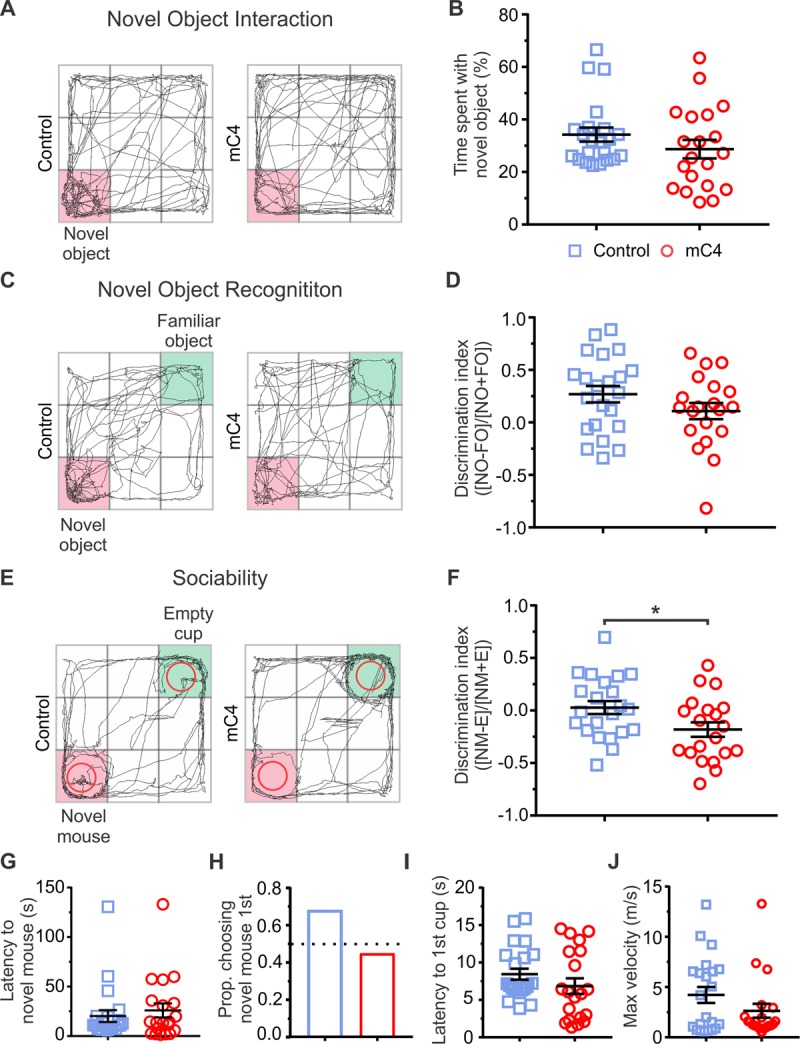
C4-dependent alterations in frontal cortical circuitry led to behavior deficits in sociability that persist into adulthood. (A) Representative examples of path traveled (black trace) by P60 control (left) and mC4 (right) adult mice in NO interaction task. Pink corner = location of NO. (B) Control and mC4 mice spent a similar amount of time exploring the NO. Percent time spent in corner with NO shown. *p* = 0.2127. (C) Representative examples of path traveled (black trace) by P60 control (left) and mC4 (right) adult mice in NO recognition task. Pink corner: location of NO. Green corner: location of FO. (D) NO recognition was intact in the mC4 condition. Control and mC4 mice had a similar DI ([time with NO − time with FO] / (time with NO + time with FO]). *p* = 0.1539. (E) Representative examples of path traveled (black trace) by P60 control (left) and mC4 (right) adult mice in sociability task. Pink corner: location of novel mouse under mesh wire cup. Green corner: location of empty mesh wire cup. (F) mC4-overexpressing mice spent less time exploring a novel mouse and more time with the empty cup (“E”) relative to control. Graph shows DI ([time with novel mouse–time with empty cup] / [time with novel mouse + time with empty cup]). *p = 0.029. (G) Control and mC4 mice had a similar latency to first approach the novel mouse (sec). *p* = 0.5416. (H) Control and mC4 mice traveled to the novel mouse cup first at a similar proportion. Proportion (“Prop.”) of mice that approached the novel mouse cup first. Fisher’s exact test. *p* = 0.54. (I) Latency to reach first cup (either novel mouse cup or empty cup) was not different (sec) between conditions. *p* = 0.217. (J) Control and mC4 mice reached the same maximum velocity (m/s). *p* = 0.1442. *N* = 22 control and *N* = 20 mC4 mice between P60 and 70. Blue: control. Red: mC4. *t* test unless otherwise stated. Mean ± SEM unless otherwise noted. For underlying data and script (respectively), see https://osf.io/7em3s/?view_only=0e7ffde4ebd344dc83af83b5a605c451 and https://github.com/balajisriram/dlc_utils. DI, discrimination index; FO, familiar object; mC4, mouse C4; NO, novel object; P, postnatal day.

Although we did not observe changes in dendritic spine density at P60 between conditions (Fig 2A), it is possible that increased C4 expression in adult mice resulted in changes in the function of frontal L2/3 neurons that could not be directly assessed by analyzing the mean dendritic spine density (e.g., miswiring of synaptic connections or intrinsic excitability). Therefore, we tested whether bilateral overexpression of mC4 in the frontal cortex led to changes in sensorimotor abilities or social behavior in adult (P60–70) mice (*N* = 22 control mice, *N* = 20 mC4 mice). We first tested adult mice in a novel-object task to assess whether interest in a novel object was affected by C4 overexpression (Fig 8A). Control and mC4 mice spent similar amounts of time exploring the novel object (Fig 8B). Next, a novel-object recognition task was performed, in which mice were placed in an arena for 5 min with a novel object in one corner and a familiar object in the opposite corner (Fig 8C). The discrimination index (DI) for object interaction (DI = [time with novel object − time with familiar object] / [time with novel object + time with familiar object]) was positive for both control and mC4 mice, indicating that mice in both conditions spent more time exploring the novel object relative to the familiar object (Fig 8D). Additionally, the DI for control and mC4 mice was not different (Fig 8D), suggesting that overexpression of mC4 in the frontal cortex did not alter sensorimotor abilities or general motivation. In support of this, mC4 mice traveled at similar speeds and distances in an open field (OF) arena relative to the control group (Figs 8J and S13).

Next, we placed either control or mC4 adult mice in an arena that contained an empty wire mesh cup and a wire mesh cup containing a novel mouse (strain, sex, and age matched) in opposing corners, and the behavior of each mouse was monitored for 5 min (Fig 8E). We found that adult mC4 mice showed a significant reduction in social DI, a metric of sociability ([time spent with novel mouse − time spent with empty cup] / [time spent with novel mouse + time spent with empty cup]), as compared to control mice (Fig 8F). This reduction in sociability was not due to an overall increase in vigilance or anxiety-like behavior in the mC4 condition, because both groups spent similar amounts of time in the open arms of the elevated-zero maze (EZM) (S13 Fig). We found no difference between control and mC4 mice in latency to approach either the novel mouse or empty cup, or in the proportion of mice choosing the novel mouse first (Fig 8G–8I). Overall, these results suggest that increased expression of mC4 in frontal L2/3 neurons led to long-lasting alterations in PFC circuitry that were sufficient to cause a reduction in social interactions in both juvenile and adult mice.

## Discussion

We showed here that overexpression of C4 in the mPFC can perturb circuit development. We demonstrated that, during normal early postnatal development, excitatory neurons in the mouse mPFC express low levels of C4. Moreover, overexpression of C4 in these cells led to spine dysgenesis that was evident through a transient decrease in filopodia and medium-sized spine density during the third week of postnatal development. We also showed that C4-dependent spine abnormalities were accompanied by a decrease in excitatory synaptic drive onto pyramidal cells in the mPFC. C4 overexpression caused microglia to engulf more PSD-95 from transfected neuron’s apical dendrites in L1, where spine loss was observed. Lastly, increased expression of C4 in the frontal cortex was sufficient to reduce social interactions in juvenile (P18) and adult (P60–70) mice. Our findings implicate C4 in shaping the developmental trajectory of cortical circuits and provide a causal link between increased C4 levels and PFC dysfunction. These data show for the first time, to our knowledge, that C4-driven circuit dysfunction in the PFC drives cellular and behavioral phenotypes observed in SCZ rather than merely being a consequence of disease and/or pharmacological treatment.

Our morphological characterization of L2/3 mPFC neurons revealed that C4-mediated connectivity deficits are both circuit and spine-type specific. That is, overexpression of C4 caused a decrease in spine density of apical but not basal dendrites of L2/3 neurons. These data provide evidence that C4-driven synaptic alterations in the mPFC are cortical layer and/or input specific. Interestingly, previous data have shown that exposure to repeated stress in adolescent rats can reduce connectivity in superficial L1 of the mPFC [47], suggesting that this layer is susceptible to genetic and environmental perturbations during development. In SCZ, dendritic spines changes are most prominent in L2/3 of the mPFC [14]; therefore, we focused on how overexpression of mC4 causes anatomical and functional changes in this region. However, imaging studies have reported structural changes in a variety of brain regions in SCZ, including the frontal and temporal lobes and hippocampus [48]. It remains unknown whether mC4 overexpression in other brain regions leads to a decrease in spine density or other abnormalities. This question could be answered by targeting other brain regions using IUE or generating a global mC4-overexpressing transgenic mouse.

When we further investigated which spine types were affected by C4 overexpression, we found a specific loss of filopodia and medium-sized spines, whereas the density of mature mushroom spines were intact. This is in line with previous observations that complement-mediated pruning is activity dependent [25]. Large mushroom and stubby spines are also associated with an accumulation of extracellular matrix proteins, which could protect them from synaptic elimination by microglia [49]. Future studies could shed light on the molecular mechanisms of input-specific plasticity by identifying “eat me”/“don’t eat me” signals [50,51] or complement proteins that are differentially present in large mushroom versus filopodia spine types.

Major contributors to synaptogenesis are dendritic filopodia, which are thin, specialized postsynaptic structures that orchestrate synapse formation by dynamically sampling potential presynaptic partners, thus optimizing circuit wiring [52,53]. It is possible that C4 contributes to aberrant synaptic wiring by reducing the ability of developing circuits to form appropriate connections. At its simplest, increased levels of C4 may underlie PFC pathology and dysfunction by specifically altering filopodia-dependent synapse formation during early development. This interpretation would suggest that subsequent aberrant synaptic elimination in SCZ is a consequence of suboptimal circuit wiring. Transient deficits in dendritic spines and filopodia are seen in several mouse models of neurodevelopmental disorders [35]. Similar to these models, our data suggest that there are sensitive developmental periods during which cortical circuitry is especially susceptible to altered expression of C4.

In our experiments, we did not observe a significant difference in apical tuft protrusion density between adult (P60) control and C4 conditions. However, in human SCZ postmortem tissue, spine loss of PFC cortical L3 neurons persists into adulthood [14]. Notably, our genetic manipulation targeted a single gene, C4, whereas SCZ results from polygenetic mechanisms [54], implicating a multitude of genes. It is possible that the mechanisms that allow for the compensation of spine density in P60 mice are also impaired in SCZ. Therefore, our data suggest that the C4-induced connectivity changes observed during the third week of development could stimulate compensatory plasticity mechanisms not present in SCZ. Additionally, there may be differences in how human and mouse pyramidal neurons respond to increased levels of C4, which could explain the discrepancy in adult dendritic spine density between mice and humans. Importantly, our qPCR and western blot data show that C4 levels at P60 were not increased in the mC4 condition mice. Therefore, the fact that spine density in C4 conditions recovered to control levels by P60 could simply be due to the lack of C4 overexpression at P60. Although spine density in C4-overexpressing neurons returned to control levels by P60, our behavioral data in adult mice suggest that the underlying circuitry remains aberrant in mice overexpressing C4 in the frontal cortex. It remains to be determined whether increased expression of C4 can lead to other abnormal changes in circuitry (i.e., L2/3 output to deeper cortical layers or PFC network properties) that were not measured in this study that persist into adulthood.

Our electrophysiological data strengthen our morphological findings and provide additional insight into the functional abnormalities caused by C4 overexpression. Previous studies have shown that microglia are able to trogocytose—or “nibble” and phagocytose—small volumes of neurons and synapses [55]. It is possible that microglia trogocytose some of the PSD without fully eliminating synapses, thus causing a reduction in synaptic strength due to a loss of glutamate receptors. In our experiments, the decrease in excitatory synaptic drive caused by C4 overexpression could be the result of an increase in phagocytosis of synapses by microglia. Alternatively, microglia trogocytosis of synaptic material could trigger synaptic elimination mediated through conventional synaptic mechanisms such as long-term depression [56]. On the other hand, C4 did not induce a change in the frequency or amplitude of mIPSCs, which is in line with previous observations showing that complement does not target GABAergic synapses in mouse and cellular models of Alzheimer’s disease [57]. The slowed kinetics of inhibitory transmission could be mediated through a loss of dendritic spines, which has been shown to alter inhibitory synapse location [58].

In addition to being the brain’s resident macrophage, microglia can influence brain development by playing roles in synapse formation, elimination, and maintenance [28,41,59]. Our data show that increased C4 expression led to morphological changes in lysosomes and enhanced phagocytic activity. Since microglia are the only cells in the brain that express CR3 [59], they are equipped to recognize material that has been tagged by the complement cascade to initiate phagocytosis. Indeed, previous work has shown that in normal brain development, microglia phagocytose synaptic material, including PSD-95, and that this process of synapse elimination is necessary for normal brain wiring [27]. Our data in the PFC are consistent with and expand upon previous studies in the retinogeniculate pathway that show microglia engulf synapses in a complement-dependent manner [16,24,25], which suggests that complement-dependent circuit wiring is necessary for normal development and contributes to pathology when mis-regulated.

Social deficits in individuals with SCZ are an early feature of this disease, and poorer social functioning is associated with worse functional outcome in adults with SCZ [60,6]. Therefore, investigating the causes of early social deficits could reveal potential targets for early therapeutic interventions in SCZ. In L1, the apical dendrites of pyramidal cells in multiple cortical layers interact with projections from higher-order areas [61]. It is thought that cortical “feedback” that inputs to L1 exerts “top-down” control that is important for higher-order cognitive processes [62]; therefore, the vulnerability of this layer to C4 overexpression could provide a cellular substrate for social dysfunction. The changes that we observed in connectivity and behavior parallel known SCZ phenotypes in humans [5,6,13–15] and provide evidence that the PFC regulates social behavior. Future studies could interrogate the role of C4 in other SCZ-relevant phenotypes such as deficits in memory function [63].

Previous work has shown that mice deficient for various complement proteins, including C3R, C1q, C3, and C4, have a reduced pruning of synaptic terminals in the visual system during development [16,24,25]. Since it has been shown that other complement proteins play a role in developmental pruning, it is possible that C4 exerts its effects through the activation of the complement cascade. However, C4 might act on other pathways in neurons to alter connectivity, independent of the complement cascade. Future work could aim to elucidate noncanonical pathways that C4 could interact with by performing proteomics or transcriptomics in neurons.

Although polygenetic mechanisms underlie SCZ [17], we provide evidence that altering expression of a single gene, C4, in mPFC neurons is sufficient to cause cellular and behavioral phenotypes that resemble SCZ pathology. We propose that mPFC neurons express low levels of C4 during normal brain development, which makes them especially susceptible to genetic and environmental risk factors that increase C4 expression, which are associated with SCZ. In summary, we have identified a critical developmental window during which prefrontal cortical circuits are susceptible to alterations in C4 expression, thus opening the possibility for early therapeutic intervention to alter the developmental trajectory of SCZ.

## Methods

### Ethics statement

All experimental protocols were conducted according to the National Institutes of Health (NIH) guidelines for animal research and were approved by the Boston University Institutional Animal Care and Use Committee (IACUC; protocol #17–031).

### Animals

All mice were group housed on a 12-hr light and dark cycle with the lights on at 7 AM and off at 7 PM and with food and water ad libitum. Offspring were housed with dams until weaning at P21. For spine density and morphology, microglia engulfment, electrophysiology, and behavior experiments, wild-type CD-1 mice of both sexes from age 7 to 70 d were used (CD-1 IGS; Charles River; strain code: 022). For in situ hybridization, *C4b* KO mice in a C57BL/6 genetic background (The Jackson Laboratory, stock number: 003643) and wild-type C57BL/6 mice of both sexes at age P30 were used (Charles River; strain code: 027). Males and females were used for all experiments, and no differences were found between sexes.

### DNA constructs

For control conditions, we used a plasmid containing EGFP under the CAG promoter (pCAG-EGFP, Addgene plasmid #11150) [64]. DNA sequences containing m*C4b* (NM_009780.2, synthesized by Genescript) and h*C4A* (RC235329, Origene) were subcloned (InFusion Kit, Clonetech) into the pCAG backbone to produce pCAG-mC4 and pCAG-hC4A, respectively. Endogenous PSD-95 was fluorescently labeled in vivo using PSD95-FingR (EF1a-PSD95.FingR-RFP) [42]. For electroporations with RFP and fusion C4-GFP, pCAG-mRFP was obtained from Addgene (Addgene plasmid #28311) [65], and C4-GFP was made by inserting GFP from the pCAG-GFP plasmid C terminus to C4 using a GSSGSS linker (subcloned by Genescript). All plasmid DNA was purified using the ZymoPureII (Zymo Research) plasmid preparation kit and were resuspended in molecular biology grade water.

### Antibodies and histology

Mice were administered a lethal dose of sodium pentobarbital (250 mg/kg; IP) before being transcardially perfused with PBS and then 4% PFA solution. After brains were dissected and postfixed for 24 hr in 4% PFA solution, they were transferred to a 30% (w/v) sucrose solution and stored at 4°C. Coronal sections were cut on a sliding microtome (Lecia SM2000) at different thicknesses appropriate for each experiment (see specific methods). For immunostaining, tissue was blocked and permeabilized in 10% donkey serum with 0.25% TritonX100. Sections were incubated with primary antibodies overnight at 4°C on a shaker and with secondary antibodies for 2 hr at room temperature. After incubation periods and between each step, sections were rinsed three times with PBS for 10 min. Depending on the experiment, the following primary antibodies were used: guinea pig anti-Iba1 (Synaptic Systems, 234004), rat anti-CD68 (Bio-Rad, MCA1957GA), rabbit anti-RFP (Rockland, 600-401-379), mouse anti-PSD95 (BioLegend, clone K28/43), guinea pig anti-Synaptophysin 1 (Synaptic Systems), rabbit anti-C4 (Biogen, 931–946), and Beta-Actin HRP-conjugate (Sigma, clone AC-15). The following secondary antibodies were used: donkey anti–guinea pig Alexa Fluor 594 (Jackson Laboratories, 706-545-148), donkey anti–guinea pig Alexa Fluor 488 (Jackson Laboratories, 706-585-148), donkey anti-rabbit Alexa Fluor 647 (Jackson Laboratories, 711-605-152), llama anti–guinea pig Atto 647 (Progen: 80308), donkey anti-rat 488 (Invitrogen: A21208), donkey anti-rabbit 594 (Jackson Laboratories, 711-585-152), and donkey anti–guinea pig 647 (Jackson Laboratories, 711-605-152). Brain sections were subsequently mounted onto microscope slides (Globe Scientific) using Fluoromount-G mounting medium with DAPI (Thermo Fisher Scientific).

### IUE

L2/3 progenitor cells in the mPFC were transfected via IUE [37]. pCAG-EGFP was electroporated in the control condition, and pCAG-EGFP plasmid was co-electroporated with either pCAG-mC4 or pCAG-hC4A plasmids for the mC4 or hC4 groups, respectively. Prior to surgery, all tools were sterilized by autoclaving. Aseptic techniques were maintained throughout the procedure, and a sterile field was prepared prior to surgery using sterile cloth drapes. Animals were weighed, and a combination of buprenorphine (3.25 mg/kg; SC) and meloxicam (1–5 mg/kg; SC) was administered as a preoperative analgesic. Timed-pregnant female CD-1 mice at E16 were anesthetized by inhalation of 4% isoflurane and maintained with 1%–1.5% isoflurane via mask inhalation. The abdomen was sterilized with 10% povidone-iodine and 70% isopropyl alcohol (repeated 3 times) before a vertical incision was made in the skin and then in the abdominal wall. The uterine horn was then exposed to allow injection of 0.5–1.0 μl of DNA solution (containing 1 μg/μl plasmid and 0.1% Fast Green) into the lateral ventricles using a pressure-injector (Picospritzer III, Parker Hannifin) with pulled-glass pipettes (Sutter Instrument, BF150-117-10). To target L2/3 progenitor cells in PFC for imaging and electrophysiological experiments, a custom-built triple electrode probe [37] was placed by the head of the embryo, with the negative electrodes placed near the lateral ventricles and the positive electrode placed just rostral of the developing PFC. For bilateral IUEs used in behavioral experiments, plasmid DNA (1 μg/μL) was injected into both lateral ventricles by positioning the glass pipette at a 90° angle relative to the midline of the embryo’s head and injecting 2–4 μl of DNA solution. These modifications ensured that the DNA solution would travel to the lateral ventricle contralateral to the injection site. Next, four square pulses (pulse duration: 50 ms, pulse amplitude: 36 V, interpulse interval: 500 ms) were delivered to the head of the embryo using a custom-built electroporator [66]. Embryos were regularly moistened with warmed sterile PBS during the surgical procedure. After electroporation, the embryos and uterine horn were gently placed back in the dam's abdominal cavity and the muscle and skin were sutured (using absorbable and nonabsorbable sutures, respectively). Finally, the dams were allowed to recover in a warm chamber for 1 hr and then returned to their cage.

### Imaging

Fluorescence images were collected using an inverted laser scanning confocal microscope (Nikon Instruments, Nikon Eclipse Ti with C2Si^+^ confocal) controlled by NisElements (Nikon Instruments, 4.51) including four laser lines (405, 488, 561, and 640 nm). For M-FISH and microglia experiments, confocal images were taken with a 60X Plan Apo objective (Nikon Instruments; Plan Apo, NA 1.4, WD: 0.14 mm, oil objective) using 1,024 × 1,024 pixel scans (pixel size = 0.27 × 0.27 μm). For dendritic spine imaging, images from P7–9, P14–16, P21–23, and P55–60 brain tissue were taken using a 40X Plan Apo λs objective (Nikon Instruments; Plan Apo, NA 1.3, WD: 0.2 mm, water objective) using 1,024 × 1,024 pixel scans (pixel resolution = 0.12). We imaged apical dendritic tufts in L1 and basal dendrites in L2/3. For dendritic spine imaging and neuronal reconstructions, we collected ROIs that consisted of stacks of images (approximately 20–40 optical sections, z-step = 0.3 μm and 1 μm, respectively). The area and diameter of neurons were analyzed in 40X images from the brightest z-plane of the soma. Brain sections from behavior mice were imaged using an upright wide-field microscope (Nikon Instruments, Nikon Eclipse Ni) controlled by NisElements (Nikon Instruments, 4.20) using a 10X objective (NA: 0.3, WD: 16 mm). DAPI and GFP-transfected cells in behavior brains were imaged using fluorescence filters for BFP (excitation: 370–401, dichroic: 420) and GFP HC (excitation: 470/40, dichroic: 495). Images in all conditions were collected using the same imaging conditions and exposure settings. Imaged stacks were imported in TIFF format into ImageJ (NIH). For confocal image analysis, we only measured neurons and glia in the anterior cingulate cortex and prelimbic, infralimbic, and medial orbital divisions of the mPFC. For behavior brain analysis, all GFP-positive cells were counted to quantify the extent of electroporation.

### Multiplex fluorescence in situ hybridization

For M-FISH experiments, P21 and P30 brains were collected and immediately fresh-frozen on dry ice in O.C.T. compound (Fisher HealthCare, 23-730-571). Tissue was cut on a cryostat (Leica CM 1800) at 15-μm thickness, and M-FISH experiments were completed by using a commercial assay (RNAscope, Advanced Cell Diagnostics). In situ fluorescent probes were used to detect mC4 (#445161-C1), EGFP (#400281-C2), and CaMKIIα (#445231-C3). M-FISH assay and all reagents were obtained from Advanced Cell Diagnostics. All M-FISH experiments had *N* = 3 mice per condition. For in situ analysis, we quantified fluorescent signal from L2/3 CaMKIIα+ cell bodies of transfected GFP+ cells and their untransfected neighbors in mC4 conditions from single z-planes of 60X confocal images. This approach allowed us to control for variability of transcript expression between mice. We use the DAPI signal to identify the cell’s nucleus, and all quantification of in situ signals was performed in the soma’s brightest focal plane. CaMKIIα signal was used to delineate the perimeter of the cell body of L2/3 excitatory neurons in the mPFC and this ROI was used for analysis. Transfected cells were identified by presence of GFP mRNA, and nontransfected cells were GFP(−). Next, fluorescent signals from GFP and C4 transcripts were thresholded and binarized. The binarized signal was then used to calculate the percentage of soma area covered by GFP or C4 mRNA. We used the same binarization threshold and analysis procedure for both conditions.

### qPCR

Animals at P21 or P60 were anesthetized with 4% isoflurane/oxygen (v/v). Brains were rapidly extracted following transcardiac perfusion using NMDG slicing solution: 92 mM NMDG, 2.5 mM KCl, 1.25 mM NaH_2_PO_4_, 30 mM NaHCO_3_, 20 mM HEPES, 25 mM glucose, 2 mM thiourea, 5 mM Na-ascorbate, 3 mM Na-pyruvate, 0.5 mM CaCl_2_·2H_2_O, and 10 mM MgSO_4_·7H_2_O. Sections (200 μm) were cut on a vibratome (LEICA VT1000 S) and kept in the NMDG slicing solution bubbled with 95% O_2_/5% CO_2_ (295–305 mOsm). The sections were trimmed under a wide-field microscope to isolate the transfected region (containing GFP+ cells) of the tissue. Each trimmed piece was flash frozen in dry ice. RNA was extracted from these tissue using Zymo Microprep kit (R1050), and cDNA was synthesized with Azuraquant cDNA synthesis kit (AZ-1997). RNA and cDNA were quantified using a Thermo Fisher Scientific NanoDrop One Spectrophotometer. Primer sets for qPCR were designed using NCBI primer blast (NIH) and were synthesized by Integrated DNA Technologies. Primer sets (Table 1) were verified to produce a single product following PCR amplification using Apex Taq RED Master Mix, 2.0X (Genesee Scientific 42–138) on a Bio-Rad T100 thermocycler. qPCR was carried out on an Applied Biosystems ABI 7900 Real Time PCR machine using AzuraView Greenfast qPCR Blue Mix HR (AZ-2401). All qPCR results were normalized to three biologically diverse internal reference genes (GAPDH, beta-Actin, and HPRT). These references were amplified in parallel with the target genes of interest and all samples were performed in triplicates. Dissociation curves were verified for every reaction, and only dissociation curves yielding single products were used in subsequent analysis.

**Table 1:** Primers used for qPCR experiments.

Gene	Primer	Sequence	Purpose
***GAPDH***	Forward	5′-CCACCCAGAAGACTGTGGAT-3′	Internal control
	Reverse	5′-CACATTGGGGGTAGGAACAC-3′	
***Beta Actin***	Forward	5′-CCTTCC TCTTGGGTATGGA-3′	Internal control
	Reverse	5′-TGCTAGGAGCCAGAGCAGTA-3′	
***HPRT***	Forward	5′-GGCCAGACTTTGTTGGATTT-3′	Internal control
	Reverse	5′-CAGATTCAACTTGCGCTCAT-3′	
***C4b***	Forward	5′-ACCCCCAGTACTTGCTGGAC-3′	Mouse C4
	Reverse	5′-ACCCTGTAGAGCAGAGCCTCTAA-3′	

Shows forward and reverse primers used in qPCR experiments (Fig 1).

Abbreviations: *GAPDH*, glyceraldehyde3-phosphate hydrogenase; *HPRT*, hypoxanthine phosphoribosyltransferase 1; qPCR, quantitative PCR.

### Cell culture

HEK293T cells were maintained in complete media: DMEM (HyClone) supplemented with 10% fetal bovine serum, penicillin (100 units/ml), and streptomycin (100 μg/ml). Cells were maintained at 37°C and 5% CO_2_ in a Thermo Scientific HERAcell 150i incubator. Either pCAG-GFP, pCAG-mC4, pCAG-hC4, or pCAG-mC4-GFP (5 μg each) was transfected into HEK293T cells (approximately 70%–80% confluent) grown in 10-cm culture dishes using 2.5 μL of GeneGlide (BioVision, Cat#: M1081) using serum-free media. After 4–6 hr, the transfection media was removed and replaced with complete media. Forty-eight hours after transfection, cells were imaged live in phenol red–free DMEM at room temperature using an Olympus BX51WI upright microscope with a 10X UPlanFL N (NA = 0.30) objective.

### Isolation of PSDs

PSD fractions were isolated as described with [57,67] minor modifications. Briefly, the electroporated region of the PFC was dissected and snap frozen and kept at −80°C until further processing. Pieces of brain tissue were homogenized using a Teflon homogenizer. After 1,400*g*, 10-min centrifugation, the supernatant was transferred to a new tube and the pellet was rehomogenized and pelleted at 1,400*g*, 10 min. Supernatants were combined and centrifugated at 13,800*g*, 10 min. The pellet was resuspended in 0.32 M Tris-buffered sucrose and ultracentrifuged into 1.2, 1.0, 0.85 M sucrose gradient at 82,500*g* for 2 hr. The synaptosome fraction was collected, solubilized with 0.5% Triton X-100, and then centrifuged at 32,800*g* for 20 min to yield the PSD fraction. Samples from individual mice were isolated separately and pooled for immunoblot analysis.

### Immunoblotting

Immunoblotting was performed as previously described [57] with minor modifications. Briefly, transfected HEK293 cells were directly lysed in the dish with 0.5% SDS in PBS, supplemented with complete protease inhibitor cocktail (Roche) and Benzonase Nuclease (Sigma). All protein samples were boiled in reducing SDS loading buffer and separated on Bolt Bis-Tris Plus gels (Thermo Fisher). After transfer to nitrocellulose membranes (Thermo Fisher), membranes were incubated with the following primary antibodies overnight: mouse anti-PSD-95 (clone K28/43, BioLegend), guinea pig anti-Synaptophysin 1 (Synaptic Systems), rabbit anti-C4 (in house, Biogen 931–946), Beta-Actin HRP-conjugate (clone AC-15, Sigma). HRP-conjugated secondary antibodies were from GE Healthcare. Immunoreactivity was detected on ChemiDoc XRS+ (Bio-Rad) and analyzed with Image Lab software. mC4 levels were quantified by normalizing to the loading control, vinculin.

### Dendritic spine analysis

For the dendritic spine developmental time-course experiments in control, mC4, and hC4 conditions, we analyzed dendritic spines at multiple postnatal days (P7–9, P14–16, P21–23, P55–60). For all groups, we analyzed 20 dendrites from 7 mice (from multiple litters). Confocal image z-stacks (40X) were background subtracted and median filtered (radius 0.25 μm), and the presence or absence of a protrusion was determined by visually inspecting the entire z-stack of images. Dendrites were selected if the entire length to be analyzed was constricted within about 15 μm in the z-plane; this typically included from the tip of the branch to the bifurcation (for apical tufts). Brightest dendrites were selected from each image to ensure that dim protrusions, such as filopodia, were reliably identified. To reliably identify spines of a given dendrite, we analyzed GFP+ dendrites that were not occluded by other nearby cell processes. Dendrite selection and all following spine analysis were conducted blindly. For a dendritic protrusion to be counted, it had to clearly protrude out of the shaft by at least 3 pixels (approximately 0.36 μm). Dendritic protrusions were quantified from either apical dendritic tufts in L1 or secondary/tertiary basal dendrites in L2/3. Spine density was calculated by dividing the number of counted spines by the total dendritic length analyzed (50–80 μm long shafts). Since GFP brightness is monotonically related to the volume in each protrusion [68], we quantified the fluorescent intensity (TIB [a.u.]) of dendritic spines in apical tufts. To obtain a TIB value, the mean gray value of the dendritic spine was measured at the brightest focal plane and was divided by the mean fluorescence intensity of the adjacent dendritic shaft to normalize for varying imaging conditions. We sorted dendritic spine types into large mushroom/stubby (TIB > 75%), medium-size spines (25% ≤ TIB ≤ 75%), and thin-spine/filopodia (TIB < 25%) intensity groups based on percentile cutoffs determined from TIB distribution values of dendritic spines. We previously showed that this is a reliable, unbiased approach for classifying dendritic spine types [69].

### Electrophysiological recordings and analysis

Mice (P18–25) were anesthetized with 4% isoflurane-oxygen mixture (v/v) and perfused intracardially with ice-cold external solution containing the following: 73 mM sucrose, 83 mM NaCl, 26.2 mM NaHCO_3_, 1 mM NaH_2_PO_4_, 22 mM glucose, 2.5 mM KCl, 3.3 mM MgSO_4_, 0.5 mM CaCl_2_ and were bubbled with 95% O_2_/5% CO_2_ (295–305 mOsm). Coronal slices (300-μm thickness) were cut on a VS1200 vibratome (Leica) in ice-cold external solution before being transferred to ACSF containing the following: 119 mM NaCl, 26 mM NaHCO_3_, 1.3 mM NaH_2_PO_4_, 20 mM glucose, 2.5 mM KCl, 2.5 mM CaCl_2_, 1.3 mM MgCl_2_, bubbled with 95% O_2_/5% CO_2_ (295–305 mOsm). Slices were kept at 35°C for 30 min before being allowed to recover for 30 min at room temperature. All recordings were performed at 30–32°C. We only recorded from transfected neurons in the anterior cingulate cortex and prelimbic, infralimbic, and medial orbital divisions of the mPFC. Signals were recorded with a 5X gain, low-pass filtered at 6 kHz, and digitized at 10 kHz using a patch-clamp amplifier (Multiclamp 700B, Molecular Devices).

Whole-cell voltage-clamp recordings were made using 3–5 MΩ pipettes filled with an internal solution that contained 125 mM Cs-gluconate, 3 mM NaCl, 8 mM CsCl, 4 mM EGTA, 4 mM MgATP, 0.3 mM NaGTP, and 10 mM HEPES (pH 7.3) with CsOH (280–290 mOsm). Series resistance (R_s_) and input resistance (R_in_) were monitored throughout the experiment by measuring the capacitive transient and steady-state deflection in response to a −5 mV test pulse, respectively. For mEPSC recordings, cells were voltage clamped at E_rev_ GABA_A_ (−70 mV) in the presence of 1 μM Tetrodotoxin (Tocris). For mIPSC recordings, cells were voltage clamped at E_rev_ Glu (+5 mV) in the presence of 1 μM Tetrodotoxin (Tocris). For all groups, we analyzed 10–12 cells from 4 mice (from multiple litters). mPSCs were detected by fitting to mPSC amplitude template using pClamp10 analysis software (Molecular Devices). The peak current of each mPSC was calculated. Next, average mPSC amplitude and IEI was calculated for each cell and then averaged across each condition to determine the population mean and SEM. Bath application of 100 μM GABA_A_ inhibitor picrotoxin at E_rev_ Glu and 20 mM CNQX and 50 mM DL-AP5 at E_rev_ GABA_A_ eliminated mPSCs, thus confirming the identity of the recorded currents. Cells were excluded if R_s_ varied by more than 20% during a recording. For all recordings, series resistance was close to 10 MΩ (control, 9.39 ± 0.53 MΩ, *n* = 12; mC4, 9.74 ± 0.44 MΩ, *n* = 10; *p* > 0.05) and was not compensated.

In current-clamp recordings, CNQX (20 mM), 50 mM DL-AP5, and picrotoxin (100 μm) were routinely added to the extracellular solution to block ionotropic synaptic transmission mediated by glutamate and GABA_A_ receptors, respectively, to assess persistent firing. Neuronal excitability was assessed using the input–output curve measured from the changes in membrane potential (presence of APs) evoked by current steps (from V_rest_, start = −200 pA, step duration: 300 ms), increasing in increments of 10–15 pA in current-clamp mode. C_m_ and R_m_ were calculated in seal-test configuration from the decay and steady-state current of a transient generated in response to a −5 mV pulse test. Rheobase is the minimum current amplitude (300 ms) that resulted in an AP.

### Neuronal morphology analysis

Confocal images of L2/3 GFP-positive neurons in mPFC (P21 and P60) were collected using a 40X objective. z-Stacks for images with a large field of view were taken at z-step of 1 μm through the entire section (about 200-μm thickness), taking care to include the entirety of the dendritic tree. Image stacks of dendritic trees and soma were imported in TIFF format into ImageJ (NIH). Cell somas and dendrites were traced manually to ensure accurate reconstruction. Dendritic arbors that could not be confidently and completely reconstructed were not used in the analysis. Dendritic reconstructions were confirmed by comparing independent reconstructions from two investigators (*N* = 10 neurons per condition). The dendritic parameters analyzed included the total dendritic length (of all dendrite branches), number of dendritic branches, number of branch points, number of dendritic end tips. Branch order for each dendritic branch was assigned starting at the cell body and increased after each branch point and the maximum branch order quantified. Sholl analysis was performed using Simple Neurite Tracer (SNT, ImageJ plugin, Longair MH, 2011). Each reconstructed neuron was thresholded and binarized, and the skeletons were imported to SNT. Sholl analysis was performed by calculating the number of dendrites that intersected concentric spheres that radiated from the soma in 10-μm radius increments. On some occasions, the dendritic arbor could not be reconstructed for several reasons: dendritic processes extended outside of the acquired field of view (or z-stack); the field of view became too dense with GFP-positive processes from neighboring cells, preventing unambiguous reconstruction; and some dendritic processes were deemed insufficiently bright to allow for unequivocal reconstruction. Thus, our reconstructions are an underestimate of the entire dendritic arbor. Neuronal soma area and diameter were measured from a single z-plane for each neuron. Soma diameter was measured perpendicular to the apical axis. Analysis was performed at P21 in the mPFC and 315 neurons were analyzed for control (6 ROIs from 3 mice) and 216 cells were analyzed for the mC4 condition (7 ROIs from 3 mice).

### Microglia assays

#### Colocalization of GFP and Iba1

GFP colocalization with microglia (Iba1) was quantified at P21. Confocal images of mPFC (60X objective) were collected using the same exposure settings between conditions including channels for DAPI, GFP (transfected neurons), and Iba1 (microglia). All images were background subtracted and binarized using the same settings between experimental groups. Analysis was completed for single z-planes. To quantify microglia proximity to GFP+ neurons, we first defined the electroporated region (ROI) to be analyzed. We created an ROI for a single z-plane in the mPFC where transfected neurons were located. There were 26 ROIs for each condition. The ROIs of electroporated regions were, on average, 98,000 μm^2^ (approximately 280 μm × 350 μm) and contained, on average, 13.6 microglia per ROI. We then identified microglia by tracing around the perimeter of the cell (using Iba1 signal), including the cell body and any proximal processes in the single analyzed z-plane. Microglia cell bodies were confirmed by the presence of DAPI. Colocalized GFP and Iba1 signal was isolated and quantified within the microglia by creating a thresholded mask. Colocalized signal within the microglia mask was only included in the analysis if it was equal to or larger than 1.23 μm (approximately 3 pixels). GFP-positive puncta within microglia were visually inspected in three-dimensional space by examining multiple z-planes to confirm the containment of the puncta to the microglia cytoplasm. The percentage of colocalized signal was quantified by dividing the area of the mask by the total area of the microglia (GFP/Iba1 colocalization [%] = microglia area colocalized with GFP / total microglia area). Using this analysis, we also calculated the percentage of microglia that were positive for GFP (GFP-positive microglia [%] = total number of GFP-positive microglia / total number of microglia), *N* = 26 ROIs (ROIs of electroporated regions from 3 mice per condition including 373 control and 334 mC4 microglia).

#### Synaptic engulfment and cortical depth correlation

To quantify engulfment of synaptic material in nonexpanded tissue, we electroporated (E16) a plasmid that labels endogenous PSD-95 (EF1a-PSD95.FingR-RFP) for control and mC4 conditions. Analysis was completed for single z-planes using DAPI, PSD-95-FingR-RFP, Iba1, and CD68 (a lysosomal marker). ROIs of electroporated regions were defined as described in the previous section (Colocalization of GFP and Iba1). Analysis was completed using the same method as in the GFP and Iba1 colocalization analysis, except synaptic material was only counted if it was within the lysosome of the microglia (PSD-95-postive puncta that colocalized with both Iba1 and CD68 signals). For each thresholded mask, the total colocalized area (microglia engulfment area [%] = microglia area colocalized with PSD-95 and CD68 / total microglia area) was quantified. We also quantified the percentage of microglia positive for engulfment (engulfment-positive microglia = [%] total number of positive microglia /total number of microglia). We also quantified the colocalization of fluorescent signals from PSD-95, Iba1, and CD68 in confocal images that were pixel shifted randomly by 12 μm in four possible directions for each channel, independently. The percent area of each microglia colocalized with CD68 signal was calculated as a measure of microglia reactivity (area of CD68 / microglia area). In a separate analysis, the cortical depth of each engulfment-positive microglia was determined by measuring the distance from the center of the cell body (using DAPI) to the adjacent pia mater. We restricted cortical depth analysis to L1 and L2/3 of the mPFC, where the dendritic spines of L2/3 cortical neurons are localized (depth ≤ 300 μm). Microglia that did not contain triple-stained puncta (PSD-95/Iba1/CD68) were excluded from correlation analysis. Control: *N* = 26 ROIs (from 5 mice; 345 microglia); mC4: *N* = 26 ROIs (from 5 mice; 319 microglia).

#### Microglia density

Microglia density analysis (P21) was completed using 50-μm z-stack images using DAPI and Iba1 signals. Microglia density was independently calculated for L1 (cortical depth: 0–120 μm) and L2/3 (cortical depth: >120–300 μm) of the mPFC. Density was calculated per ROI (electroporated region) by dividing the number of microglia in the ROI by the total volume of tissue for each ROI. Microglia density was measured in 19 control ROIs (from 5 mice, 2,146 microglia) and 17 mC4 ROIs (from 5 mice, 1,640 microglia) in the superficial layers of cortex, and in each ROI the density was calculated for L1 and L2/3. Microglia cell bodies were confirmed by the presence of DAPI.

### ExM

Coronal mouse brain sections (50 μm) were permeabilized as previously described and stained at 4°C for 24 hr with primary antibodies (rat anti-CD68, rabbit anti-RFP, guinea pig anti-Iba1) at a dilution of 1:500 for each. Tissue was subsequently washed three times for 10 min in 1X PBS with 0.025% Triton-100X. The tissue was then incubated with secondary antibodies (anti-rat 488, anti-rabbit Alexa 546, anti-rabbit 594, anti–guinea pig Atto 647, anti–guinea pig Alexa 647) at 4°C for 24 hr. Tissue sections were again washed three times with PBS for 10 min. Sections were then anchored in 0.1 mg/ml Acryloyl-X SE in 1X PBS for 12 hr and polymerized within a polyacrylamide and sodium acrylate gel [70]. The gels were trimmed and subsequently digested with proteinase k (8 units/ml) for 8–10 hr. Following digestion, the sections were transferred into a #1.5 glass-bottom dish, washed three times with 1X PBS, and expanded with milliQ purified water for 15 min twice. Fluorescence images of the expanded tissue were acquired with a Nikon C2Si inverted laser scanning confocal microscope (Nikon Instruments, Nikon Eclipse Ti with C2Si+ confocal) with a 40X Plan Apo λs objective (Nikon Instruments; Plan Apo, NA 1.3, WD: 0.2 mm, water objective). Images of microglia near apical dendrites of L2/3 FingR-PSD95-RFP(+) neurons were acquired with a pixel sampling of (0.155 μm/pixel) and 1-μm z-axis step interval.

The scaling factor for each expanded microglia was determined by comparing pre-expansion measurements of the long and short cross sections of the microglial cell body in a maximum-intensity projection image, and these values were compared to expanded microglia measurements. The scaling factor for each microglia was calculated based on pre-expansion images. This scaling factor was applied to any measurements made within that expanded image (e,g., lysosomes area) to calculate the original size of the organelles. To determine the number and size of lysosomes, ROIs were manually drawn following the perimeter of the lysosome using the z-axis plane with the brightest CD68 signal. This z-plane was determined first qualitatively and confirmed by a line scan through the approximate center of the lysosome signal, which yielded the largest slope of intensity versus distance. The lysosome ROIs were counted and the cross-sectional areas measured and compared between experimental groups. CD68-positive signals were scored as a lysosome if they were 3 × 3 pixels, or 7–9 clustered pixels (0.216 μm^2^ expanded), in size with an average signal intensity of at least two times above local background levels and were present in two consecutive z-planes.

Additionally, each lysosomal ROI was verified for FingR-PSD95-RFP signal using a z-axis profile plot for both CD68 and RFP signal. ROIs that show overlapping peaks between RFP and CD68 were verified for signal within the ROI in comparison to the surrounding local background. Local background was determined by calculating the mean background signal of RFP in a region surrounding each ROI. We considered lysosomes positive for synaptic engulfment if the ROI signal increased by two standard deviations over the local background signal. PSD95-FIngR-RFP-positive signal was scored as a PSD-95 internalized puncta if they were 2 × 2 pixels, or 4–5 clustered pixels (0.0961 μm^2^ expanded), in size with an average signal intensity of at least two times above local background levels. The data were derived from 5 and 4 mice each for control and mC4 conditions, respectively, and 45 and 41 microglia were analyzed from control and mC4 conditions, respectively. We analyzed a total of 1,987 and 2,531 lysosomes from control and mC4 conditions, respectively.

### MI task

For all juvenile behavior tasks, we used control (*N* = 15) and mC4 (*N* = 21) mice that were electroporated bilaterally at E16. Prior to the maternal homing test, dams were acclimated to the behavioral task by placing them in the mesh cup for 5-min periods, for 3 d consecutively. Mice were separated from their dams for 1 hr immediately before testing. The MI task consisted of two phases. In phase 1 (MI1), mice were placed in an OF with home bedding and fresh bedding, and in phase 2 (MI2) mice were placed in an OF with home bedding, and their dam was restrained in a small wire mesh cup and an empty wire mesh cup. Both phases of the behavioral task were run on P18.

For MI1, individual pups were transferred to a homemade acrylic arena (50 × 50 × 30 cm length-width-height) and placed in a “starting corner.” The arena contained fresh bedding in two neutral corners and nest bedding in the corner opposite from the starting corner. Mice explored for 3 min, and the total time spent in the starting, nest, and fresh corners was measured using a homemade video tracking system written in MATLAB (MathWorks). Grooming occurrences and time spent grooming were annotated by a trained experimenter blind to experimental conditions.

For MI2, two wire mesh cups were placed in opposite corners of a homemade acrylic arena (50 × 50 × 30 cm length-width-height), one containing the animal's dam and the other empty, and the start corner contained soiled home bedding. Mice spent 5 min exploring the MI2 environment, and we video-recorded behavior using a Logitech C270 Webcam at 30 fps. Time spent near each cup and other areas was measured using our homemade video tracking system that tracked the centroid of the mouse. Behaviors were recorded under a dim light (approximately 20 lux) positioned over the center zone of the arena. Between trials and mice, the arena was cleaned with 70% ethanol.

### Adult behavior

For all adult behavior tasks, we used control (*N* = 22) and mC4 (*N* = 20) mice that were electroporated bilaterally at E16. The same cohort of adult mice were run in all adult behavioral tasks between the ages of P60–70. All behavior was recorded using a Logitech C270 Webcam at 20 fps. All mice were handled for 3 d consecutively prior to any behavioral testing to ensure familiarity to the experimenter. For all behavioral tasks, experiments were recorded under a dim light (approximately 20 lux) positioned over the center zone of the arena. For OF, mice were placed in a homemade acrylic arena (50 × 50 × 30 cm length-width-height) and were free to explore for 5 min. For EZM, mice were placed in the closed arm of a homemade EZM (track diameter = 50 cm, track width = 5 cm, wall height for closed arms = 40 cm, height of track = 61 cm) and were free to explore for 5 min. For OF and EZM, mice were not exposed to the arenas until testing day, since the initial response to the environment is measured. Sociability and object tasks were run in the OF arena. Mice were acclimated to the OF arena for 3 d prior to behavioral testing (5 min each day). For the novel-object interaction task, mice were placed in the center of the arena that contained a novel object (small plastic toy with smooth, cleanable surfaces) in one corner (corner alternated between mice). Mice were free to explore the arena for 5 min. For novel-object recognition, the arena contained a familiar object (object exposed in novel-object task) in one corner and a novel object in the opposite corner (corners alternated between mice), and mice were free to explore for 5 min. For the sociability task, the arena contained a novel mouse (age, sex, and strain matched) under a mesh wire cup in one corner and an empty mesh wire cup in the opposing corner, and mice were free to explore for 5 min. For all behavioral tasks, the arena was cleaned with 70% ethanol between all trials.

All adult behavior was analyzed using DeepLabCut [71], an open-source software package that uses deep neural networks to automatically track body parts from videos. We confirmed accurate tracking of mice based on this software by close inspection of videos once they had been annotated by DeepLabCut. For all adult behavior position tracking, we used the centroid of the mouse and compared amount of time spent in ROIs. For OF, we calculated the maximum and average velocity and the total distance traveled. For EZM, we compared the time spent in the open arms of the maze. For both novel-object and sociability tasks, we quantified amount of time spent in the corners of interest in the arena (time spent with novel object for novel-object interaction, time spent with novel versus familiar object for novel-object recognition, time spent with novel mouse versus empty cup for sociability).

### Quantification of behavior brains

We counted the number of GFP-positive cells and assessed the extent of cell transfection in brains of animals that were tested in behavioral tasks. Experimental groups included mice from at least two litters (*N* = 15 mice for control and *N* = 21 for mC4 condition for juvenile behaviors; *N* = 22 mice for control and *N* = 20 for mC4 condition for adult behaviors). Somas were counted by multiple trained, independent experimenters. Coronal sections (50-μm thickness) from the entire brain were carefully inspected, and the DAPI signal was used to corroborate the presence of cell nuclei. We used a brain atlas [72] to confirm the location of GFP+ cell bodies and to delineate the boundaries of each brain region. Most transfected cells were located in frontal cortical regions, between Bregma +3.0 mm and 1.6 mm. We did not observe transfected cells in caudal cortical regions or subcortical areas.

To estimate the total amount of cells transfected in each brain, cells were counted from 50-μm sections, including every other section. Since transfections were homogenous across neighboring brain sections, once we obtained the total number of cells per section, we plotted cell counts against their respective Bregma coordinates. We then used a linear interpolation to estimate the cell counts from missing brain sections. In a small number of brains (*N* = 3), we found no differences in total cell counts when we compared values obtained from counting GFP-positive cells in all transfected brain sections to estimated values obtained using the interpolation approach. In total, including juvenile and adult brains, we counted 174,041 neurons from 78 brains. All control and mC4 brains from mice used in behavioral tasks were GFP+ and had similar rostro-caudal patterns of transfected neurons. We did not find any differences in total cell counts between groups for juvenile or adult conditions.

### Statistical analysis

For confocal image analysis and electrophysiological recordings, we focused on neurons in the anterior cingulate cortex and prelimbic, infralimbic, and medial orbital divisions of the mPFC. All statistical analysis was completed in GraphPad Prism 8.0, and threshold for significance for all tests was set to 0.05 (α = 0.05). M-FISH and qPCR data sets were analyzed with an unpaired *t* test. Spine developmental data were analyzed using a one- or two-way ANOVA followed by Tukey’s posttest. Dendritic spine fluorescent intensity values were sorted based off of percentile cutoffs determined from TIB distribution values of dendritic spines in control conditions, and differences in the density of spine types between groups were tested with a one-way ANOVA followed by Tukey’s posttest. Electrophysiological data were analyzed with an unpaired *t* test, and cumulative distributions were analyzed with a Kolmogorov-Smirnov (KS) test. Microglia data were analyzed using either an unpaired *t* test or a one-way ANOVA followed by Tukey’s posttest. Behavioral data were analyzed with a two-way ANOVA followed by Sidak’s posttest and a *t* test with Welch’s correction. Dendrite and soma morphology measurements were analyzed using a *t* test. Both male and female mice were used, and we did not observe any differences between the sexes among groups. Correlations were determined using Pearson’s r correlation and linear regression. Analysis was performed blind to condition. Soma area/diameter and dendrite measurements were analyzed using GraphPad Prism 8.0 (GraphPad Software). Figures were prepared using CorelDRAW Graphics Suite X8 (Corel Corporation) and ImageJ (NIH). Custom-written routines for behavioral tracking and analysis are available upon request. Data are presented as the mean ± SEM, unless otherwise noted.

## Supporting information

S1 Fig IUE increased amount of mC4 transcript in neurons. (A) Representative 60X confocal images of in situ hybridization showing that C4b mRNA is expressed in mPFC superficial layers in P30 WT mice. White dotted line: pia mater. White circles: nuclei with C4 mRNA. (B) Representative 60X confocal images of in situ hybridization showing that C4b mRNA was not expressed in mPFC superficial layers in P30 C4b KO mice. White dotted line: pia mater. (A-B) Confirmed in 3 mice per condition. Scale bar = 60 μm. (C) Representative 60X confocal images at P21 of in situ hybridization from the same coronal section showing CaMKIIα+ neurons that were transfected with GFP and mC4 (C4b) (white arrowhead) and untransfected neighbors expressing mC4 (pink arrowhead). Scale bar = 15 μm. (D) IUE reliably increased C4b transcript levels in transfected cells. Percent of soma area positive for C4 transcript in transfected and untransfected neurons. *N* = 100 neurons (3 mice) per condition. *t* test. ****p < 0.0001. Mean ± SEM. (E) Transcript levels of GFP and mC4 positively correlated in transfected cells. Black line: linear fit. Gray lines: 95% confidence intervals. Blue dotted line: average endogenous C4 expression at P21 in CaMKIIα+ mPFC L2/3 neurons. *N* = 100 transfected neurons (3 mice). Pearson’s r correlation and linear regression. r = 0.28. ****p < 0.0001. For underlying data, see https://osf.io/7em3s/?view_only=0e7ffde4ebd344dc83af83b5a605c451. CaMKIIα, calcium/calmodulin-dependent protein kinase type II subunit alpha; GFP, green fluorescent protein; IUE, in utero electroporation; KO, knock-out; L, layer; mC4, mouse C4; mPFC, medial prefrontal cortex; P, postnatal day; WT, wild-type.
(TIF)

S2 Fig PSD synaptosome isolation from PFC tissue. (A) Immunoblot assay showing C4 staining with anti-C4 antibody (clone 931–946). Samples were from HEK293 cells transfected with GFP, hC4A, mC4, or mC4-GFP constructs. We detected C4 variants as approximately 250-kDa proteins, which is likely the unprocessed protein (predicted molecular weight is 193 kDa) or not fully reduced C4 protein or not fully reduced C4 proteint eurons? Also did you ever compare neuronal expression vs astrocytes vs microglia?ssion in WT a (C4 has disulfide bonds). (B) Fractionation scheme for the preparation of PSDs from mouse PFC region. Fractions that were used for immunoblot analysis are shown in bold. (C) Immunoblot of postsynaptic (PSD-95) and presynaptic (synaptophysin 1) marker proteins in PSD isolation steps. Synaptosome fraction contains both PSD-95 and synaptophysin 1. For underlying data, see https://osf.io/7em3s/?view_only=0e7ffde4ebd344dc83af83b5a605c451. GFP, green fluorescent protein; hC4A, human C4A; HEK, human epithelial kidney; mC4, mouse C4; PFC, prefrontal cortex; PSD, postsynaptic density.
(TIF)

S3 Fig mC4-GFP protein is expressed in transfected HEK cells and L2/3 neurons. (A) Representative 10X wide-field images of HEK cells transfected with either GFP (top panels) or fusion mC4-GFP (bottom panels). Left panel shows brightfield image. Middle and right panels show GFP signal (cyan). Right panel is zoom region of yellow square in middle panel. Scale bar left and middle panels = 100 μm. Scale bar right panels = 25 μm. (B) Representative 40X confocal image of IUE-transfected L2/3 neurons in the mPFC of P21 mice. Neurons cotransfected with pCAG-RFP (magenta) and pCAG-mC4-GFP (cyan). Bottom panels are zoomed region from the yellow square in the top left panel. Yellow arrowheads in bottom panels show C4-GFP signal in the dendrites of a neuron. Scale bar top panels = 50 μm. Scale bar bottom panels = 15 μm. GFP, green fluorescent protein; HEK, human epithelial kidney; IUE, in utero electroporation; L, layer; mC4, mouse C4; mPFC, medial prefrontal cortex; P, postnatal day; RFP, red fluorescent protein.
(TIF)

S4 Fig Overexpression of mC4 did not alter soma size or proximal dendrite width. (A) Soma area was not different between control and mC4 conditions. *t* test. *p* = 0.13. (B) mC4 overexpression did not alter the diameter of neurons. *t* test. *p* = 0.37. (A-B) Only GFP-positive L2/3 mPFC neurons included in analysis. Data points represent average measures from ROIs containing many neurons from 3 mice per condition. Control: *N* = 6 ROIs (including 316 neurons). mC4: *N* = 7 ROIs (including 216 neurons). (C) Primary dendrite width was not different between conditions. *N* = 10 neurons per condition. Data points represent average primary dendrite width per neuron, including all primary apical and basal dendrites. *t* test. *p* = 0.16. Mean ± SEM. For underlying data, see https://osf.io/7em3s/?view_only=0e7ffde4ebd344dc83af83b5a605c451. GFP, green fluorescent protein; L, layer; mC4, mouse C4; mPFC, medial prefrontal cortex; ROI, region of interest.
(TIF)

S5 Fig Dendrite morphology was not altered by mC4 overexpression at P21. (A) Representative confocal images (40X) of control and mC4 GFP-positive L2/3 neurons in the mPFC at P21. Images are max z-projections. Scale bar = 50 μm. (B) Reconstructions of control and mC4 neurons from (A). Black lines: apical dendrites. Gray lines: basal dendrites. Red line: axon. Light blue: cell body. Scale bar = 50 μm. (C) There was no difference in total dendritic length (μm) between control and mC4 neurons. *t* test. *p* = 0.59. (D) There was no difference in maximum branch order between control and mC4 neurons. *t* test. *p* = 0.44. (E) mC4 overexpression did not change the total number of branches in PFC L2/3 neurons. *t* test. *p* = 0.54. (F) There was no difference in the total number of dendritic end tips between control and mC4 neurons. *t* test. *p* = 0.44. (G) There was no difference in total number of branch points between conditions. *t* test. *p* = 0.29. (H) No difference found in the sum of Sholl intersections between control and mC4 neurons. *t* test. *p* = 0.41. (I) Number of intersections as a function of Sholl radii (μm). Dark blue line: control mean. mC4, dark red line: mC4 mean. Light blue shade: control SEM. Light red shade: mC4 SEM. (C-I) *N* = 10 neurons per condition. Blue data points: control. Red data points: mC4. Mean ± SEM. For underlying data, see https://osf.io/7em3s/?view_only=0e7ffde4ebd344dc83af83b5a605c451. GFP, green fluorescent protein; L, layer; mC4, mouse C4; mPFC, medial prefrontal cortex; P, postnatal day.
(TIF)

S6 Fig Microglia were in closer proximity to neurons overexpressing mC4 at P21. (A) Representative single z-plane confocal image (60X) showing microglia (“MG”) interacting with processes of electroporated neurons in superficial layers of mPFC (left). White dotted line: pia. Left scale bar = 50 μm. Higher magnification of insets (left) of L1 and L2/3. White arrowhead: microglia (MG). Right scale bar = 10 μm. (B) Representative confocal image (60X) showing microglia (Iba1, magenta) colocalized with neuronal GFP signal in P21 histological sections for control (top) and mC4 (bottom) conditions. White arrow heads: Iba1/GFP-positive puncta colocalized with microglia soma. Left: max z-projection of entire microglia; scale bar: 7 μm. Right: single z-plane; scale bar = 3.5 μm. (C) Overexpression of mC4 in L2/3 neurons increased the number of microglia that colocalized with GFP+ neuronal material (GFP-positive microglia [%]). *t* test. ****p < 0.0001. (D) mC4 overexpression increased the percentage microglia area colocalized with neuronal material (MG area [%] = area of MG GFP+ / total MG area). *t* test. ***p = 0.0009. (C-D) *N* = 26 ROIs (transfected region) from 3 mice per condition (including 373 control and 334 mC4 microglia). Mean ± SEM. For underlying data, see https://osf.io/7em3s/?view_only=0e7ffde4ebd344dc83af83b5a605c451. GFP, green fluorescent protein; Iba1, ionized calcium binding adaptor molecule 1; L, layer; mC4, mouse C4; mPFC, medial prefrontal cortex; P, postnatal day; ROI, region of interest.
(TIF)

S7 Fig Microglia density and lysosome size were not altered by mC4 overexpression. (A) Microglia (“MG”) density in superficial layers of the mPFC was not affected by mC4 overexpression. Control: *N* = 19 ROIs (from 5 mice including 2,146 microglia). mC4: *N* = 17 ROIs (from 5 mice including 1,640 microglia). One-way ANOVA with Bonferroni’s multiple comparisons. *p* = 0.998. (B) Microglia lysosomal areas, as measured by area of microglia positive for CD68, were not different between conditions. Area of MG CD68+ (%) = area of microglial CD68+ / total microglia area. Control: *N* = 26 ROIs (from 5 mice including 345 microglia). mC4: *N =* 26 ROIs (from 5 mice including 319 microglia). *t* test. *p* = 0.19. Mean ± SEM. For underlying data, see https://osf.io/7em3s/?view_only=0e7ffde4ebd344dc83af83b5a605c451. mC4, mouse C4; mPFC, medial prefrontal cortex; ROI, region of interest.
(TIF)

S8 Fig Intrabodies against PSD95 label excitatory synapses of L2/3 pyramidal neurons. (A) Representative confocal image (60X) showing cytoarchitecture (DAPI, blue) and labeling of endogenous PSD-95 by FingR (PSD95-FingR-RFP, pseudocolored green) in P21 coronal sections. Scale bar = 25 μm. (B) PSD95-FingR labeling pattern was consistent with endogenous location of synaptic PSD-95 in L2/3 pyramidal neurons. Mean normalized fluorescent intensity (normalized to peak PSD95-FingR signal) as a function of distance from pia (μm). Dark blue line: mean. Light blue shade: SEM. Bar graph: mean normalized fluorescent intensity (y-axis) is greater in L1 (yellow inset) than in L4 (red inset). *t* test. *p* < 0.0001. (C) Zoomed images from image in (A) showing boxed regions in L1 (yellow inset) and L4 (red inset). White arrowheads: PSD95-FingR puncta. Blue: DAPI (cell nuclei). Scale bar = 5 μm. For underlying data, see https://osf.io/7em3s/?view_only=0e7ffde4ebd344dc83af83b5a605c451. L, layer; P, postnatal day; PSD, postsynaptic density.
(TIF)

S9 Fig ExM expanded microglia by approximately 2.8x. (A) Representative photograph of a coronal PFC brain section before (left) and after (right) expansion. Scale bar = 1 cm. (B) Representative confocal image (40X) of a lysosome (CD68+) before (left) and after (right) expansion. Scale bar = 1 μm. (C) ExM revealed greater detail of lysosome morphology. Mean gray value line intensity scan (for dotted lines shown in B). Blue line: pre-expansion. Yellow line: post-expansion. (D) Representative confocal image (40X) of an Iba+ microglia before (left) and after (right) expansion. Scale bar = 5 μm. (E) Graph showing the long diameter of each microglia before (blue) and after (red) expansion and after post-scaling (purple). One-way ANOVA with Tukey’s. ****p < 0.0001. (F) Graph showing the short diameter of each microglia before (blue) and after (red) expansion and after post-scaling (purple). One-way ANOVA with Tukey’s. ****p < 0.0001. (E-F) The average scaling factor was approximately 2.78 (control: 2.79 ± 0.069; mC4: 2.76 ± 0.092). *N* = 86 microglia (45 control microglia and 41 mC4 microglia). Post-scaled = post / scaling factor (2.78). Inset image of microglia shows the long (E) and short (F) diameter of soma (white line with arrows). The coefficient of variation between pre-expansion, post-expansion, and post-expansion scaled microglia sizes was 30.24%, 34.35%, and 29.22%, respectively. The variance between the pre-expansion and post-expansion scaled sizes was compared with an F-test, and no difference was found between these populations (one-way ANOVA with Tukey’s test; *p* = 0.4212). Mean ± SEM. For underlying data, see https://osf.io/7em3s/?view_only=0e7ffde4ebd344dc83af83b5a605c451. ExM, expansion microscopy; Iba, ionized calcium binding adaptor molecule 1; PFC, prefrontal cortex.
(TIF)

S10 Fig ExM revealed that neuronal mC4 overexpression increased the number of PSD-95+ lysosomes in microglia. (A-B) Representative confocal images (40X) of a single expanded microglia from control (A) and mC4 (B) conditions, respectively. The columns in (A) and (B) show different z-planes of a single microglia in each of the columns that contain lysosomes that were either positive (+) or negative (−) for PSD-95. These images show that mC4 condition microglia contained a greater number of lysosomes positive for PSD-95. The first row shows microglia (Iba1) with a silhouette drawn in white. The second row shows lysosomes (CD68) within the microglia shown in the top panel. The bottom two rows are a zoomed region (yellow inset from row 2) showing lysosomes (third row) and PSD-95 (fourth row) with a silhouette of the lysosome drawn in white. White arrowheads show PSD-95 within lysosomes. Scale bar (A) = 1 μm. Scale bar (B) = 1.63 μm. ExM, expansion microscopy; Iba1, ionized calcium binding adaptor molecule 1; mC4, mouse C4; PSD, postsynaptic density.
(TIF)

S11 Fig Targeting large populations of L2/3 frontal cortex neurons using IUE. (A) Percentage of GFP+ cells per area in juvenile (P18) mC4 mice. *N* = 21 mC4 mice. (B) Total number of GFP+ cells for juvenile mice for control and mC4. *N* = 36 mice (15 control and 21 mC4 mice). (C) Percentage of GFP+ cells per area in adult mC4 mice. *N* = 20 mC4 mice. (D) Total number of GFP-positive cells per area for adult mice (P60) for control and mC4. *N* = 42 mice (22 control and 20 mC4). (E) Representative sections showing rostro-caudal extent of transfections in the frontal cortex. Images in left panels are zoomed areas from the right panels (red square). Black numbers: Bregma coordinates. Left panel scale bar = 0.5 mm. Right panel scale bar = 1 mm. Mean ± SEM. For underlying data, see https://osf.io/7em3s/?view_only=0e7ffde4ebd344dc83af83b5a605c451. ACC, anterior cingulate cortex; AI, anterior insular cortex; AO, anterior olfactory nucleus; Cpu, caudate-putamen; Fr3, frontal cortex area 3; FrA, frontal association cortex; GFP, green fluorescent protein; IL, infralimbic cortex; L, layer; LO, lateral orbitofrontal cortex; M1, primary motor cortex; M2, supplementary motor cortex; mC4, mouse C4; MO, medial orbitofrontal cortex; P, postnatal day; Pir, piriform cortex; PL, prelimbic cortex; S1, primary somatosensory cortex; VO, ventral orbitofrontal cortex.
(TIF)

S12 Fig mC4 pups had increased grooming occurrences and grooming time compared to controls. (A) mC4 mice had a greater number of grooming occurrences during the MI1 task. *t* test with Welch’s correction. *p < 0.05. (B) Average time per each grooming occurrence was longer for mC4 mice compared to controls in the MI1 task. *t* test with Welch’s correction. **p < 0.01. (A-B) *N* = 36 mice (15 control and 21 mC4). Mean ± SEM. For underlying data, see https://osf.io/7em3s/?view_only=0e7ffde4ebd344dc83af83b5a605c451. M2, supplementary motor cortex; mC4, mouse C4; MI1, maternal interaction 1.
(TIF)

S13 Fig Adult mice bilaterally expressing mC4 had intact motor abilities. (A) Control and mC4 mice (P60) had a similar average velocity in the OF task. *t* test. *p* = 0.2141. (B) Control and mC4 mice traveled similar total distances in the OF task. *t* test. *p* = 0.1986. (C) Control and mC4 mice spent a similar amount of time exploring the open arms of the EZM task. *t* test. *p* = 0.0651. (A-C) *N* = 42 mice (22 control and 20 mC4 mice). Mean ± SEM. For underlying data, see https://osf.io/7em3s/?view_only=0e7ffde4ebd344dc83af83b5a605c451. EZM, elevated-zero maze; mC4, mouse C4; OF, open field; P, postnatal day.
(TIF)

## Abbreviations

AP: action potential
*C4* KO: *C4b* knock-out
CR3: complement 3 receptor
CaMKIIα: calcium/calmodulin-dependent protein kinase type II subunit alpha
DI: discrimination index
E: embryonic day
ExM: expansion microscopy
EZM: elevated-zero maze
GFP: green fluorescent protein
h*C4*: human *C4*
HEK: human epithelial kidney
IEI: interevent interval
IUE: in utero electroporation
Iba1: ionized calcium binding adaptor molecule 1
L: layer
m*C4*: mouse *C4*
mEPSC: miniature excitatory postsynaptic current
M-FISH: multiplex in situ hybridization
MHC: major histocompatibility complex
MI1: maternal interaction 1
MI2: maternal interaction 2
mIPSC: miniature inhibitory postsynaptic current
mPFC: medial prefrontal cortex
OF: open field
P: postnatal day
PFC: prefrontal cortex
PSD: postsynaptic density
qPCR: quantitative PCR
RFP: red fluorescent protein
SCZ: schizophrenia
TIB: total integrated brightness
