# Supplementary figures and images for: Increased expression of schizophrenia-associated gene C4 leads to hypoconnectivity of prefrontal cortex and reduced social interaction
Source: PLoS Biol. 2020 Jan 14;18(1):e3000604. doi: 10.1371/journal.pbio.3000604 (PMC6959572; doi:10.1371/journal.pbio.3000604)

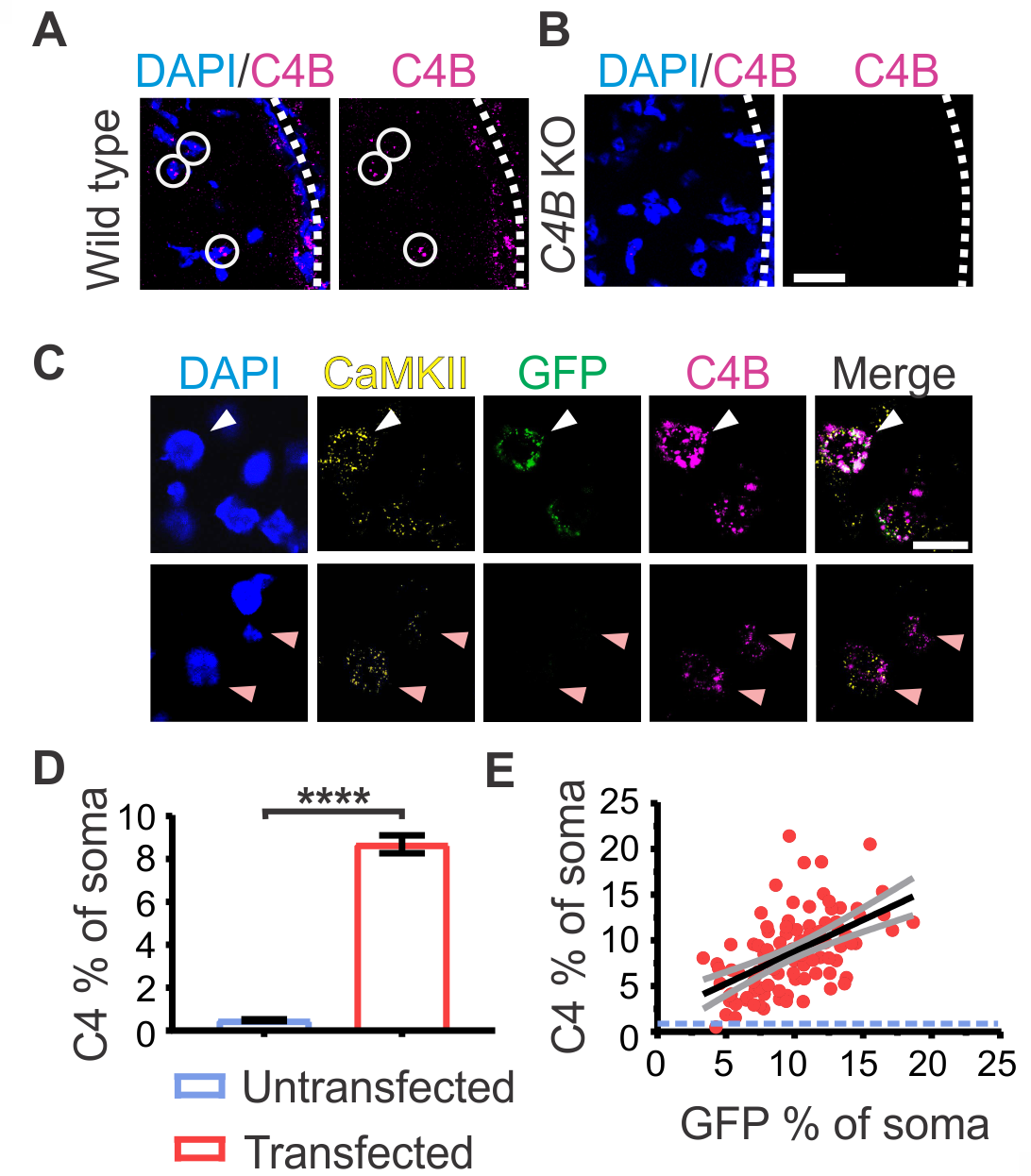

Supplement: S1 Fig — (A) Representative 60X confocal images of in situ hybridization showing that C4b mRNA is expressed in mPFC superficial layers in P30 WT mice. White dotted line: pia mater. White circles: nuclei with C4 mRNA. (B) Representative 60X confocal images of in situ hybridization showing that C4b mRNA was not expressed in mPFC superficial layers in P30 C4b KO mice. White dotted line: pia mater. (A-B) Confirmed in 3 mice per condition. Scale bar = 60 μm. (C) Representative 60X confocal images at P21 of in situ hybridization from the same coronal section showing CaMKIIα+ neurons that were transfected with GFP and mC4 (C4b) (white arrowhead) and untransfected neighbors expressing mC4 (pink arrowhead). Scale bar = 15 μm. (D) IUE reliably increased C4b transcript levels in transfected cells. Percent of soma area positive for C4 transcript in transfected and untransfected neurons. N = 100 neurons (3 mice) per condition. t test. ****p < 0.0001. Mean ± SEM. (E) Transcript levels of GFP and mC4 positively correlated in transfected cells. Black line: linear fit. Gray lines: 95% confidence intervals. Blue dotted line: average endogenous C4 expression at P21 in CaMKIIα+ mPFC L2/3 neurons. N = 100 transfected neurons (3 mice). Pearson’s r correlation and linear regression. r = 0.28. ****p < 0.0001. For underlying data, see https://osf.io/7em3s/?view_only=0e7ffde4ebd344dc83af83b5a605c451. CaMKIIα, calcium/calmodulin-dependent protein kinase type II subunit alpha; GFP, green fluorescent protein; IUE, in utero electroporation; KO, knock-out; L, layer; mC4, mouse C4; mPFC, medial prefrontal cortex; P, postnatal day; WT, wild-type. (TIF) [file pbio.3000604.s001.tif]

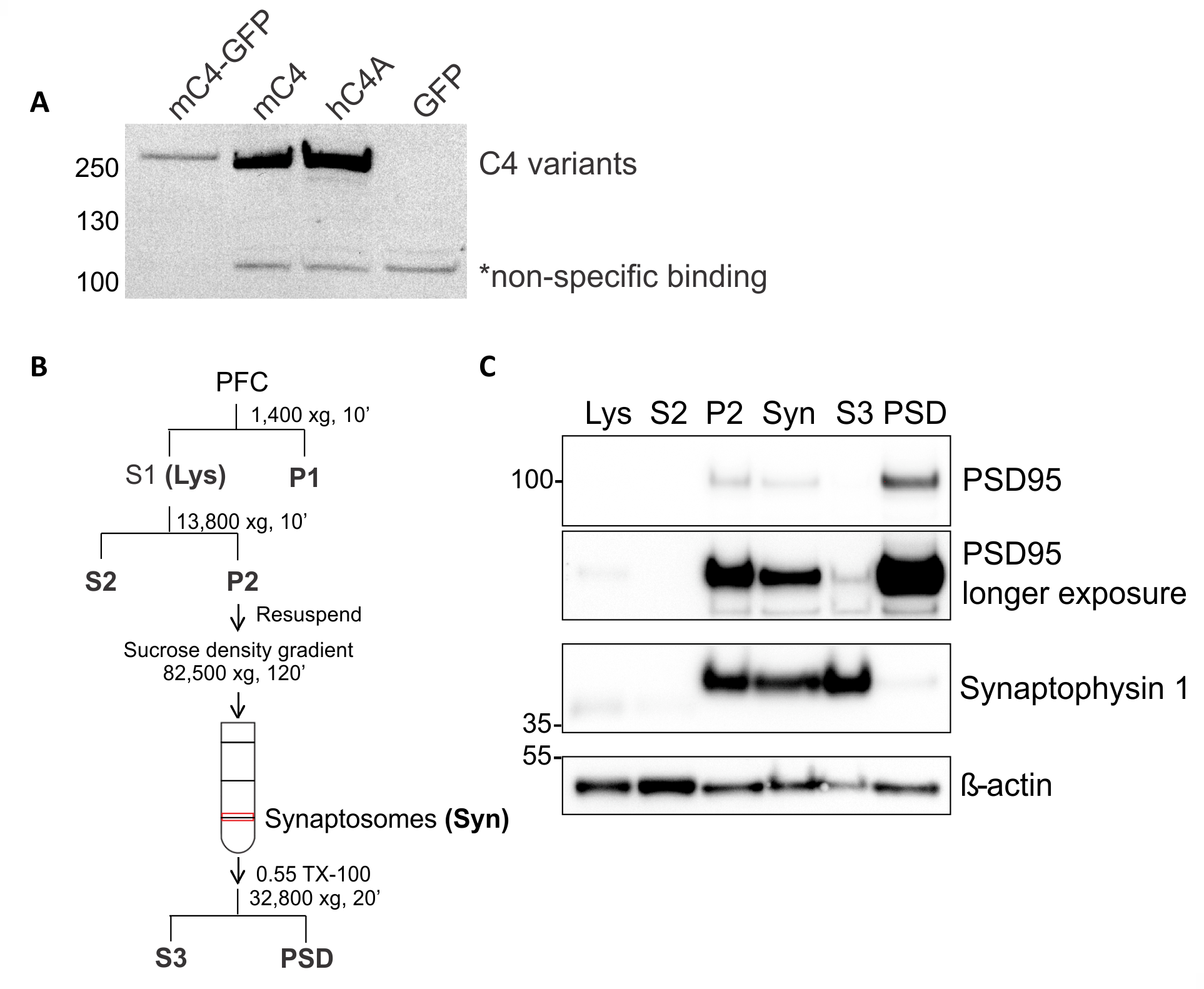

Supplement: S2 Fig — (A) Immunoblot assay showing C4 staining with anti-C4 antibody (clone 931–946). Samples were from HEK293 cells transfected with GFP, hC4A, mC4, or mC4-GFP constructs. We detected C4 variants as approximately 250-kDa proteins, which is likely the unprocessed protein (predicted molecular weight is 193 kDa) or not fully reduced C4 protein or not fully reduced C4 proteint eurons? Also did you ever compare neuronal expression vs astrocytes vs microglia?ssion in WT a (C4 has disulfide bonds). (B) Fractionation scheme for the preparation of PSDs from mouse PFC region. Fractions that were used for immunoblot analysis are shown in bold. (C) Immunoblot of postsynaptic (PSD-95) and presynaptic (synaptophysin 1) marker proteins in PSD isolation steps. Synaptosome fraction contains both PSD-95 and synaptophysin 1. For underlying data, see https://osf.io/7em3s/?view_only=0e7ffde4ebd344dc83af83b5a605c451. GFP, green fluorescent protein; hC4A, human C4A; HEK, human epithelial kidney; mC4, mouse C4; PFC, prefrontal cortex; PSD, postsynaptic density. (TIF) [file pbio.3000604.s002.tif]

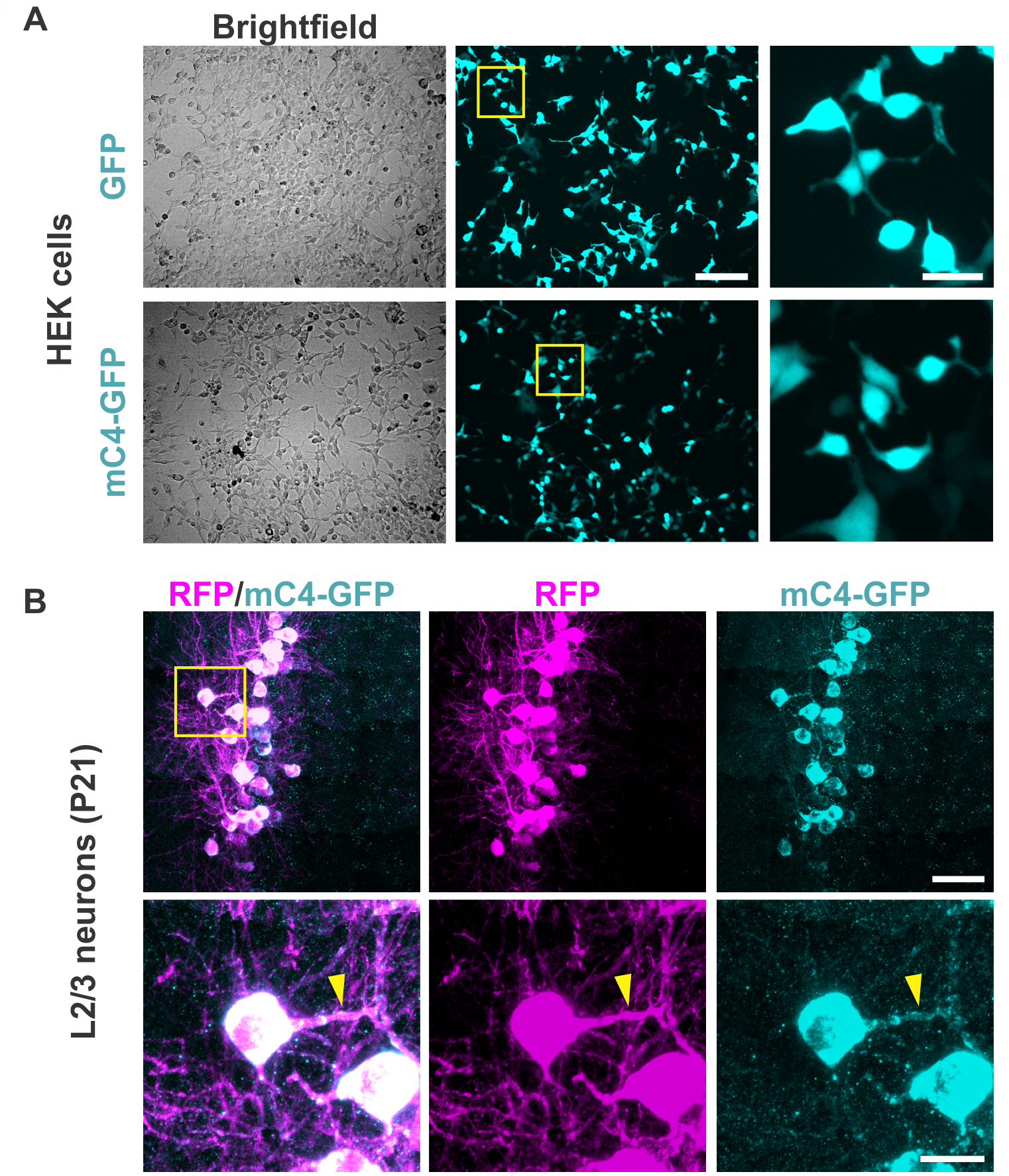

Supplement: S3 Fig — (A) Representative 10X wide-field images of HEK cells transfected with either GFP (top panels) or fusion mC4-GFP (bottom panels). Left panel shows brightfield image. Middle and right panels show GFP signal (cyan). Right panel is zoom region of yellow square in middle panel. Scale bar left and middle panels = 100 μm. Scale bar right panels = 25 μm. (B) Representative 40X confocal image of IUE-transfected L2/3 neurons in the mPFC of P21 mice. Neurons cotransfected with pCAG-RFP (magenta) and pCAG-mC4-GFP (cyan). Bottom panels are zoomed region from the yellow square in the top left panel. Yellow arrowheads in bottom panels show C4-GFP signal in the dendrites of a neuron. Scale bar top panels = 50 μm. Scale bar bottom panels = 15 μm. GFP, green fluorescent protein; HEK, human epithelial kidney; IUE, in utero electroporation; L, layer; mC4, mouse C4; mPFC, medial prefrontal cortex; P, postnatal day; RFP, red fluorescent protein. (TIF) [file pbio.3000604.s003.tif]

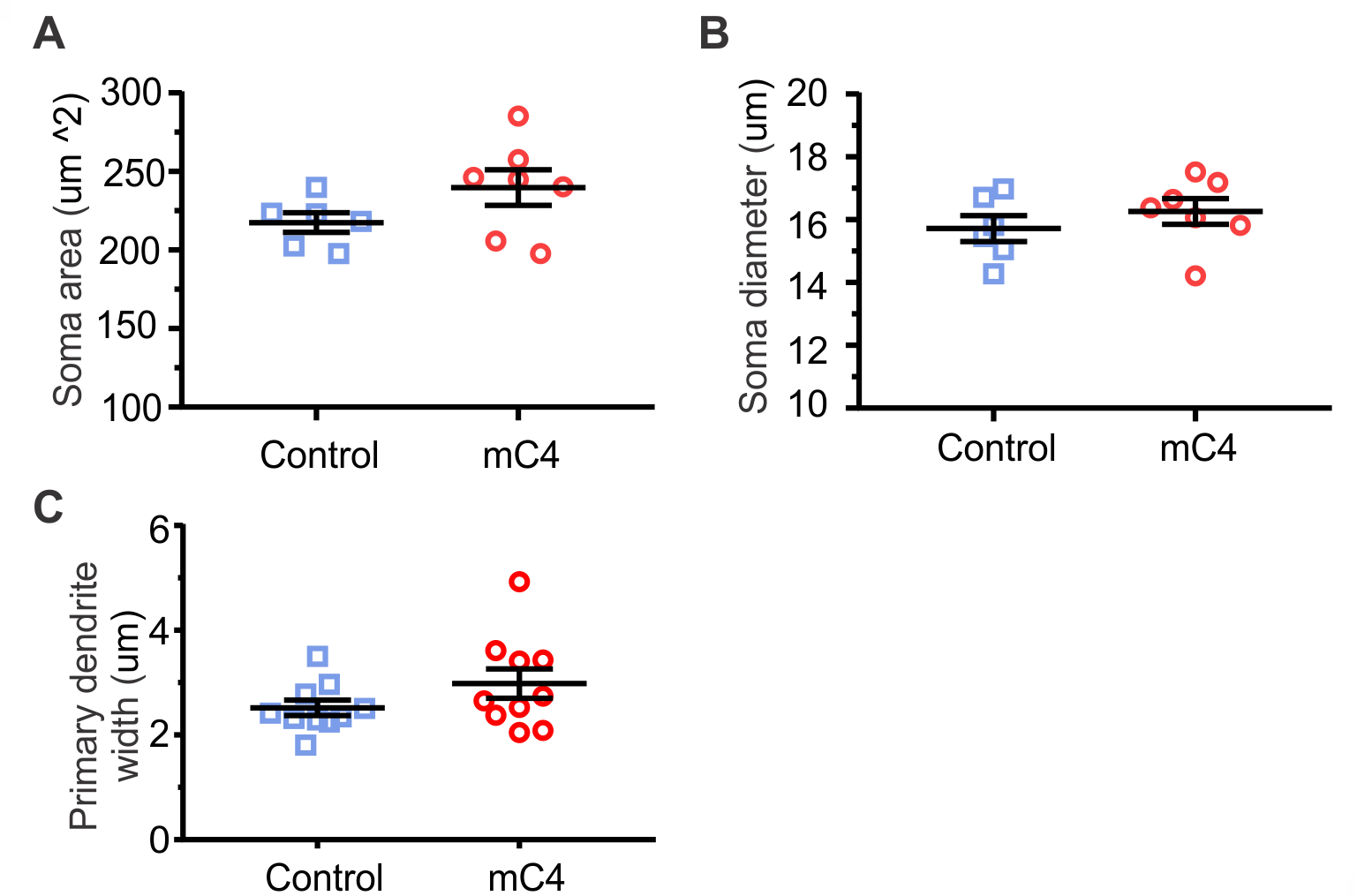

Supplement: S4 Fig — (A) Soma area was not different between control and mC4 conditions. t test. p = 0.13. (B) mC4 overexpression did not alter the diameter of neurons. t test. p = 0.37. (A-B) Only GFP-positive L2/3 mPFC neurons included in analysis. Data points represent average measures from ROIs containing many neurons from 3 mice per condition. Control: N = 6 ROIs (including 316 neurons). mC4: N = 7 ROIs (including 216 neurons). (C) Primary dendrite width was not different between conditions. N = 10 neurons per condition. Data points represent average primary dendrite width per neuron, including all primary apical and basal dendrites. t test. p = 0.16. Mean ± SEM. For underlying data, see https://osf.io/7em3s/?view_only=0e7ffde4ebd344dc83af83b5a605c451. GFP, green fluorescent protein; L, layer; mC4, mouse C4; mPFC, medial prefrontal cortex; ROI, region of interest. (TIF) [file pbio.3000604.s004.tif]

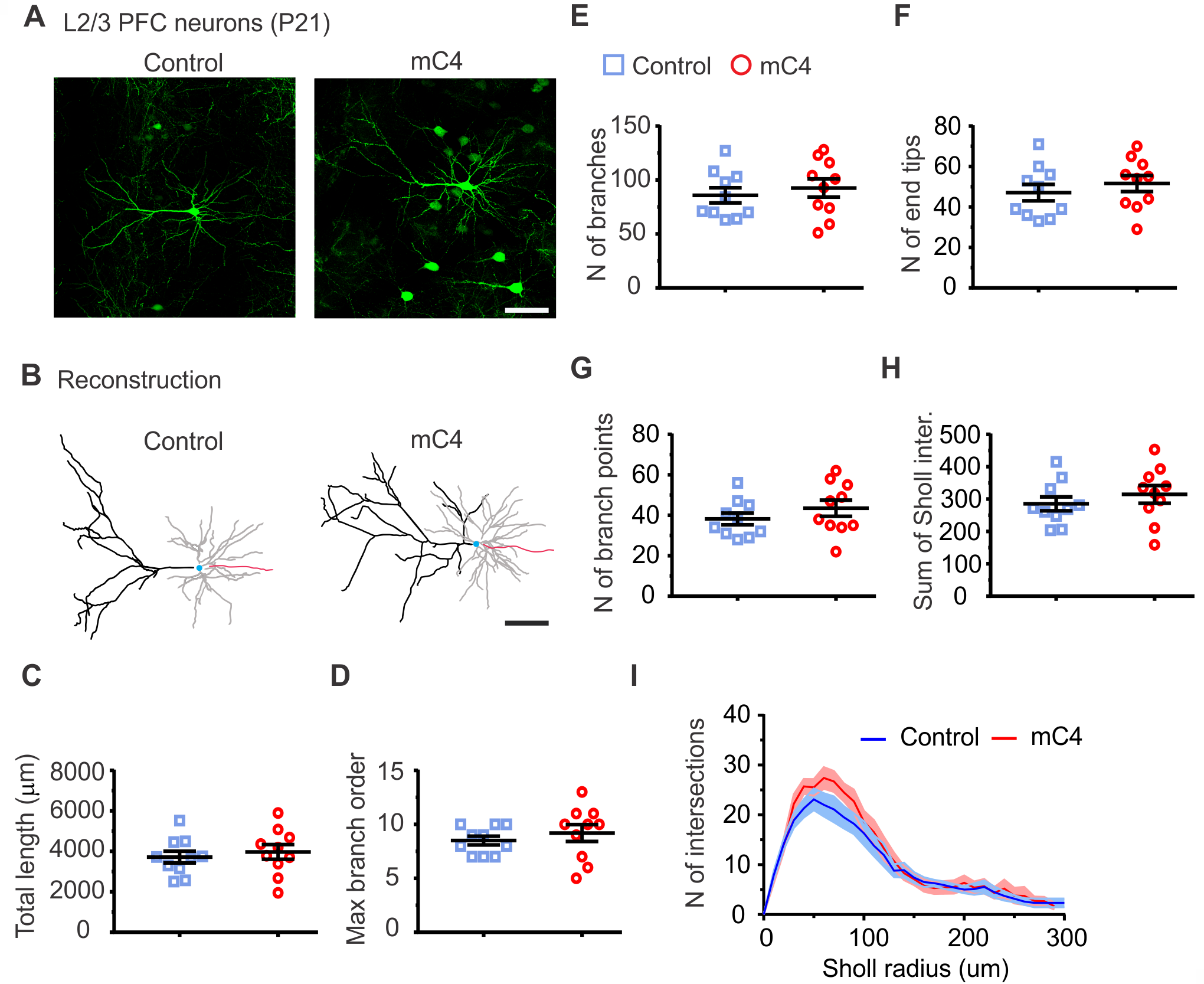

Supplement: S5 Fig — (A) Representative confocal images (40X) of control and mC4 GFP-positive L2/3 neurons in the mPFC at P21. Images are max z-projections. Scale bar = 50 μm. (B) Reconstructions of control and mC4 neurons from (A). Black lines: apical dendrites. Gray lines: basal dendrites. Red line: axon. Light blue: cell body. Scale bar = 50 μm. (C) There was no difference in total dendritic length (μm) between control and mC4 neurons. t test. p = 0.59. (D) There was no difference in maximum branch order between control and mC4 neurons. t test. p = 0.44. (E) mC4 overexpression did not change the total number of branches in PFC L2/3 neurons. t test. p = 0.54. (F) There was no difference in the total number of dendritic end tips between control and mC4 neurons. t test. p = 0.44. (G) There was no difference in total number of branch points between conditions. t test. p = 0.29. (H) No difference found in the sum of Sholl intersections between control and mC4 neurons. t test. p = 0.41. (I) Number of intersections as a function of Sholl radii (μm). Dark blue line: control mean. mC4, dark red line: mC4 mean. Light blue shade: control SEM. Light red shade: mC4 SEM. (C-I) N = 10 neurons per condition. Blue data points: control. Red data points: mC4. Mean ± SEM. For underlying data, see https://osf.io/7em3s/?view_only=0e7ffde4ebd344dc83af83b5a605c451. GFP, green fluorescent protein; L, layer; mC4, mouse C4; mPFC, medial prefrontal cortex; P, postnatal day. (TIF) [file pbio.3000604.s005.tif]

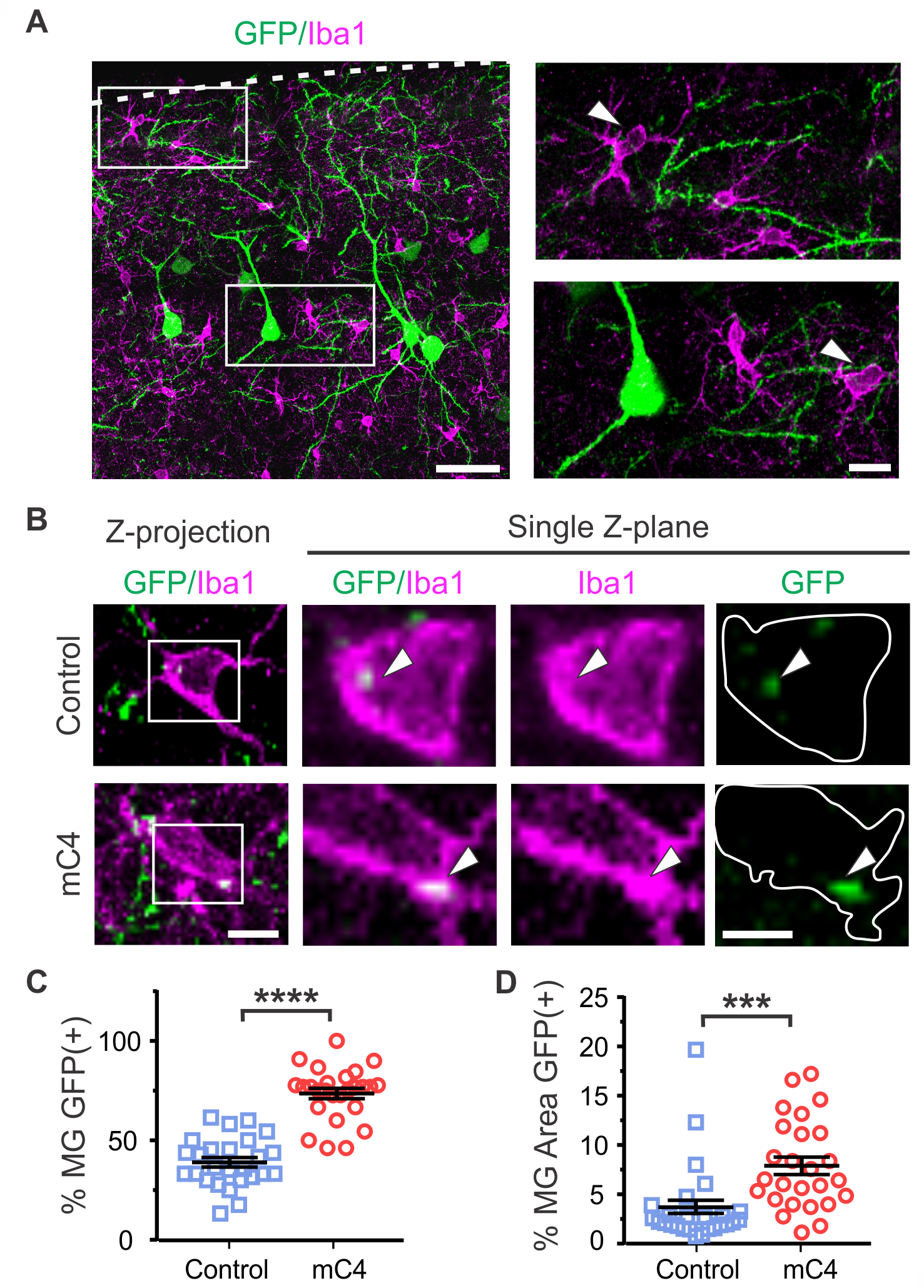

Supplement: S6 Fig — (A) Representative single z-plane confocal image (60X) showing microglia (“MG”) interacting with processes of electroporated neurons in superficial layers of mPFC (left). White dotted line: pia. Left scale bar = 50 μm. Higher magnification of insets (left) of L1 and L2/3. White arrowhead: microglia (MG). Right scale bar = 10 μm. (B) Representative confocal image (60X) showing microglia (Iba1, magenta) colocalized with neuronal GFP signal in P21 histological sections for control (top) and mC4 (bottom) conditions. White arrow heads: Iba1/GFP-positive puncta colocalized with microglia soma. Left: max z-projection of entire microglia; scale bar: 7 μm. Right: single z-plane; scale bar = 3.5 μm. (C) Overexpression of mC4 in L2/3 neurons increased the number of microglia that colocalized with GFP+ neuronal material (GFP-positive microglia [%]). t test. ****p < 0.0001. (D) mC4 overexpression increased the percentage microglia area colocalized with neuronal material (MG area [%] = area of MG GFP+ / total MG area). t test. ***p = 0.0009. (C-D) N = 26 ROIs (transfected region) from 3 mice per condition (including 373 control and 334 mC4 microglia). Mean ± SEM. For underlying data, see https://osf.io/7em3s/?view_only=0e7ffde4ebd344dc83af83b5a605c451. GFP, green fluorescent protein; Iba1, ionized calcium binding adaptor molecule 1; L, layer; mC4, mouse C4; mPFC, medial prefrontal cortex; P, postnatal day; ROI, region of interest. (TIF) [file pbio.3000604.s006.tif]

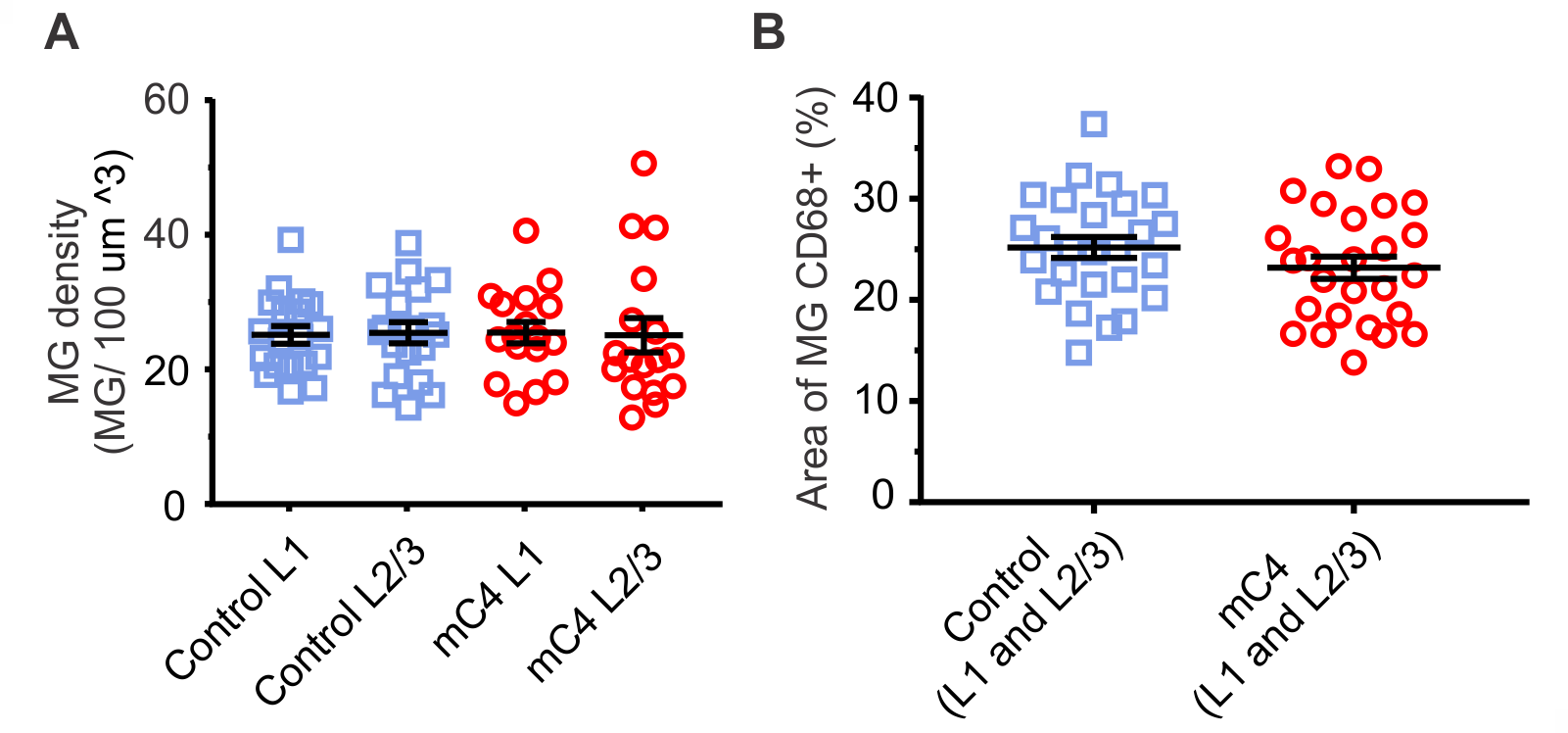

Supplement: S7 Fig — (A) Microglia (“MG”) density in superficial layers of the mPFC was not affected by mC4 overexpression. Control: N = 19 ROIs (from 5 mice including 2,146 microglia). mC4: N = 17 ROIs (from 5 mice including 1,640 microglia). One-way ANOVA with Bonferroni’s multiple comparisons. p = 0.998. (B) Microglia lysosomal areas, as measured by area of microglia positive for CD68, were not different between conditions. Area of MG CD68+ (%) = area of microglial CD68+ / total microglia area. Control: N = 26 ROIs (from 5 mice including 345 microglia). mC4: N = 26 ROIs (from 5 mice including 319 microglia). t test. p = 0.19. Mean ± SEM. For underlying data, see https://osf.io/7em3s/?view_only=0e7ffde4ebd344dc83af83b5a605c451. mC4, mouse C4; mPFC, medial prefrontal cortex; ROI, region of interest. (TIF) [file pbio.3000604.s007.tif]

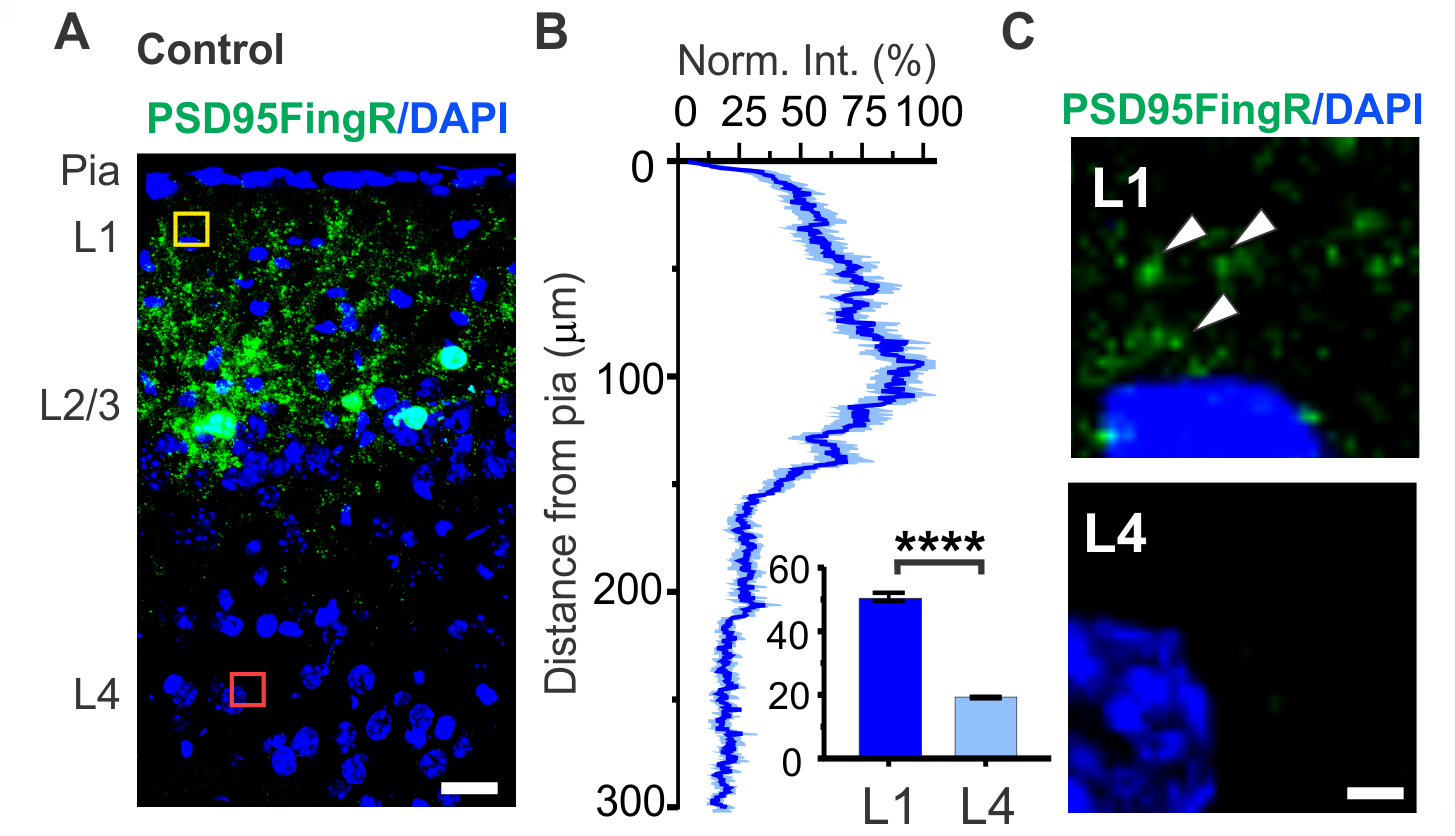

Supplement: S8 Fig — (A) Representative confocal image (60X) showing cytoarchitecture (DAPI, blue) and labeling of endogenous PSD-95 by FingR (PSD95-FingR-RFP, pseudocolored green) in P21 coronal sections. Scale bar = 25 μm. (B) PSD95-FingR labeling pattern was consistent with endogenous location of synaptic PSD-95 in L2/3 pyramidal neurons. Mean normalized fluorescent intensity (normalized to peak PSD95-FingR signal) as a function of distance from pia (μm). Dark blue line: mean. Light blue shade: SEM. Bar graph: mean normalized fluorescent intensity (y-axis) is greater in L1 (yellow inset) than in L4 (red inset). t test. p < 0.0001. (C) Zoomed images from image in (A) showing boxed regions in L1 (yellow inset) and L4 (red inset). White arrowheads: PSD95-FingR puncta. Blue: DAPI (cell nuclei). Scale bar = 5 μm. For underlying data, see https://osf.io/7em3s/?view_only=0e7ffde4ebd344dc83af83b5a605c451. L, layer; P, postnatal day; PSD, postsynaptic density. (TIF) [file pbio.3000604.s008.tif]

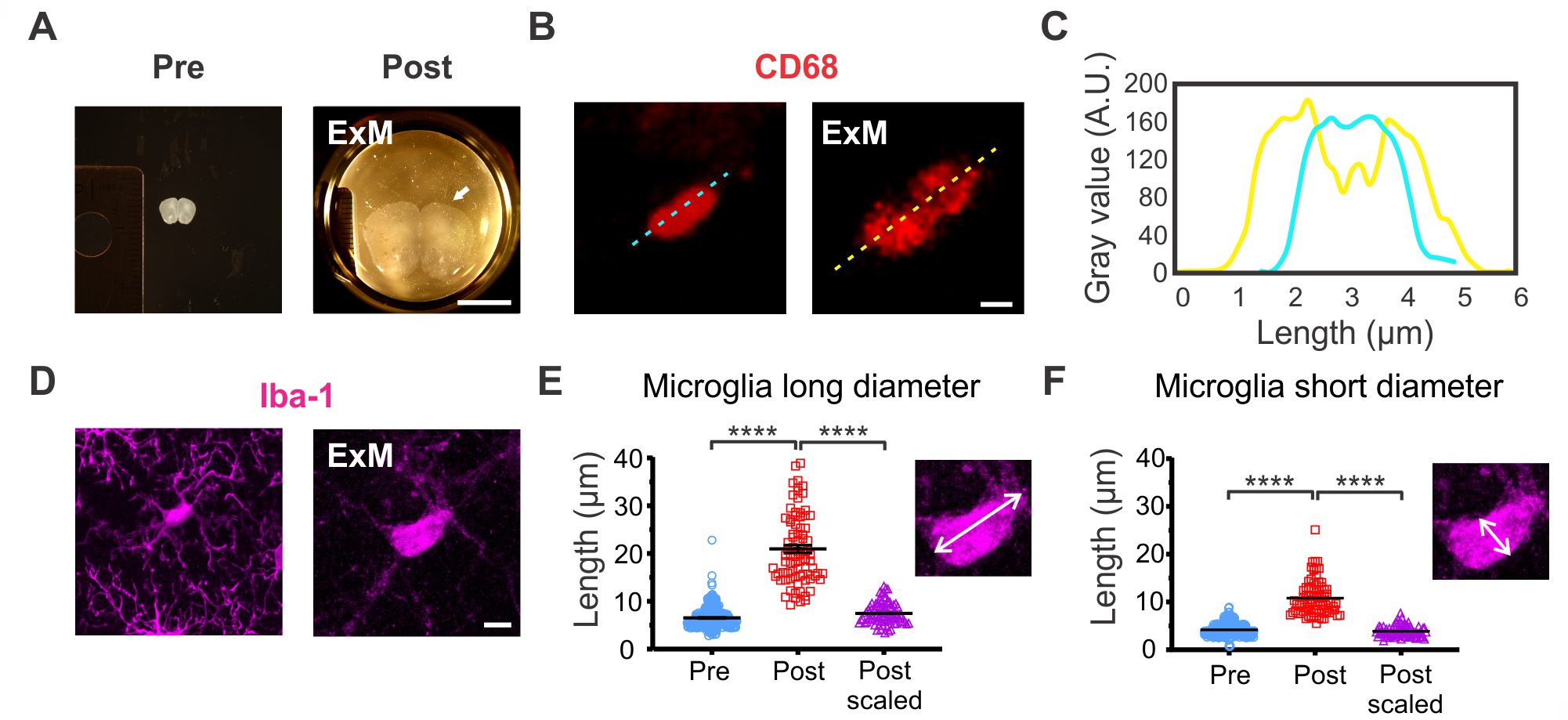

Supplement: S9 Fig — (A) Representative photograph of a coronal PFC brain section before (left) and after (right) expansion. Scale bar = 1 cm. (B) Representative confocal image (40X) of a lysosome (CD68+) before (left) and after (right) expansion. Scale bar = 1 μm. (C) ExM revealed greater detail of lysosome morphology. Mean gray value line intensity scan (for dotted lines shown in B). Blue line: pre-expansion. Yellow line: post-expansion. (D) Representative confocal image (40X) of an Iba+ microglia before (left) and after (right) expansion. Scale bar = 5 μm. (E) Graph showing the long diameter of each microglia before (blue) and after (red) expansion and after post-scaling (purple). One-way ANOVA with Tukey’s. ****p < 0.0001. (F) Graph showing the short diameter of each microglia before (blue) and after (red) expansion and after post-scaling (purple). One-way ANOVA with Tukey’s. ****p < 0.0001. (E-F) The average scaling factor was approximately 2.78 (control: 2.79 ± 0.069; mC4: 2.76 ± 0.092). N = 86 microglia (45 control microglia and 41 mC4 microglia). Post-scaled = post / scaling factor (2.78). Inset image of microglia shows the long (E) and short (F) diameter of soma (white line with arrows). The coefficient of variation between pre-expansion, post-expansion, and post-expansion scaled microglia sizes was 30.24%, 34.35%, and 29.22%, respectively. The variance between the pre-expansion and post-expansion scaled sizes was compared with an F-test, and no difference was found between these populations (one-way ANOVA with Tukey’s test; p = 0.4212). Mean ± SEM. For underlying data, see https://osf.io/7em3s/?view_only=0e7ffde4ebd344dc83af83b5a605c451. ExM, expansion microscopy; Iba, ionized calcium binding adaptor molecule 1; PFC, prefrontal cortex. (TIF) [file pbio.3000604.s009.tif]

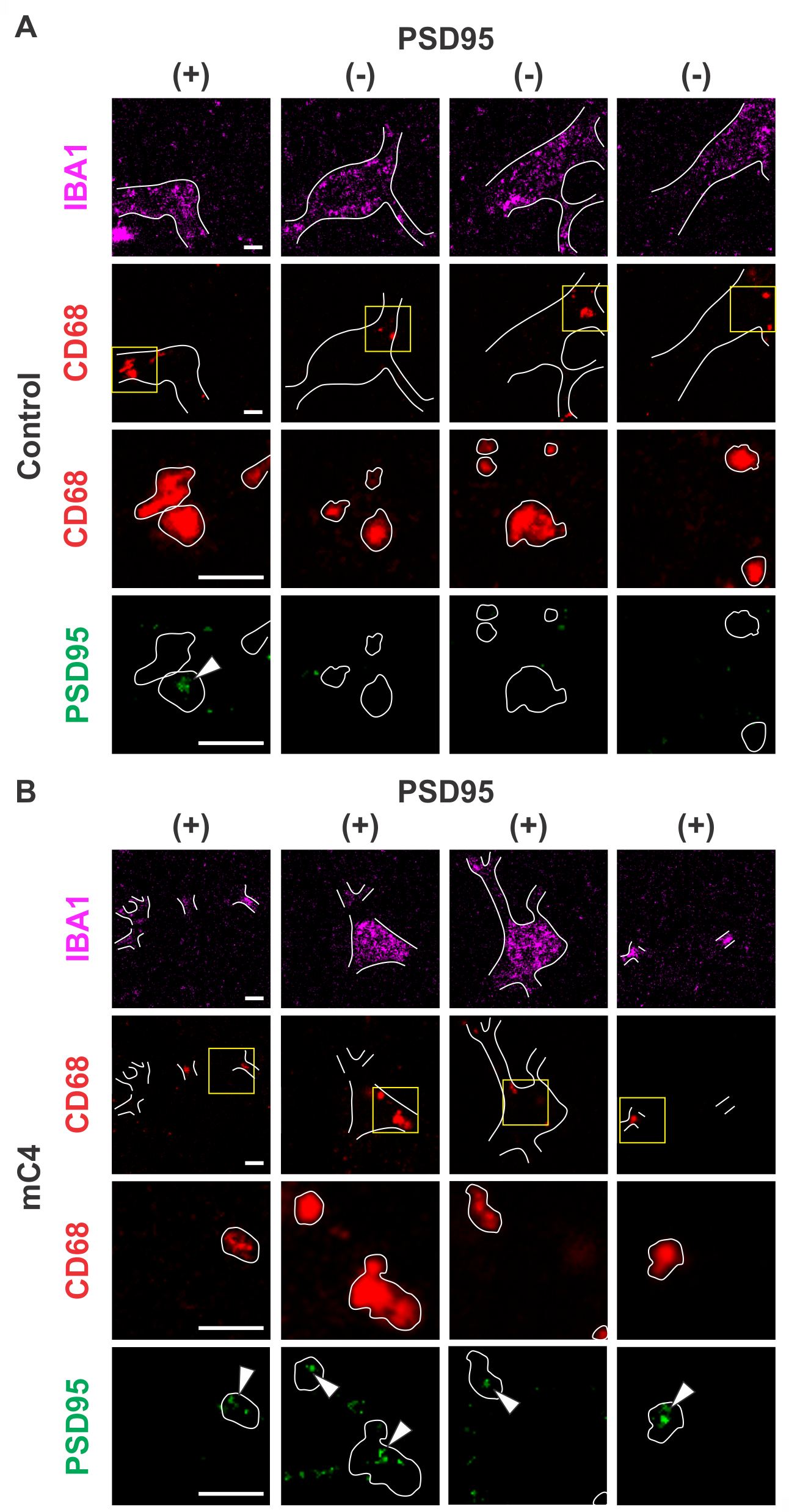

Supplement: S10 Fig — (A-B) Representative confocal images (40X) of a single expanded microglia from control (A) and mC4 (B) conditions, respectively. The columns in (A) and (B) show different z-planes of a single microglia in each of the columns that contain lysosomes that were either positive (+) or negative (−) for PSD-95. These images show that mC4 condition microglia contained a greater number of lysosomes positive for PSD-95. The first row shows microglia (Iba1) with a silhouette drawn in white. The second row shows lysosomes (CD68) within the microglia shown in the top panel. The bottom two rows are a zoomed region (yellow inset from row 2) showing lysosomes (third row) and PSD-95 (fourth row) with a silhouette of the lysosome drawn in white. White arrowheads show PSD-95 within lysosomes. Scale bar (A) = 1 μm. Scale bar (B) = 1.63 μm. ExM, expansion microscopy; Iba1, ionized calcium binding adaptor molecule 1; mC4, mouse C4; PSD, postsynaptic density. (TIF) [file pbio.3000604.s010.tif]

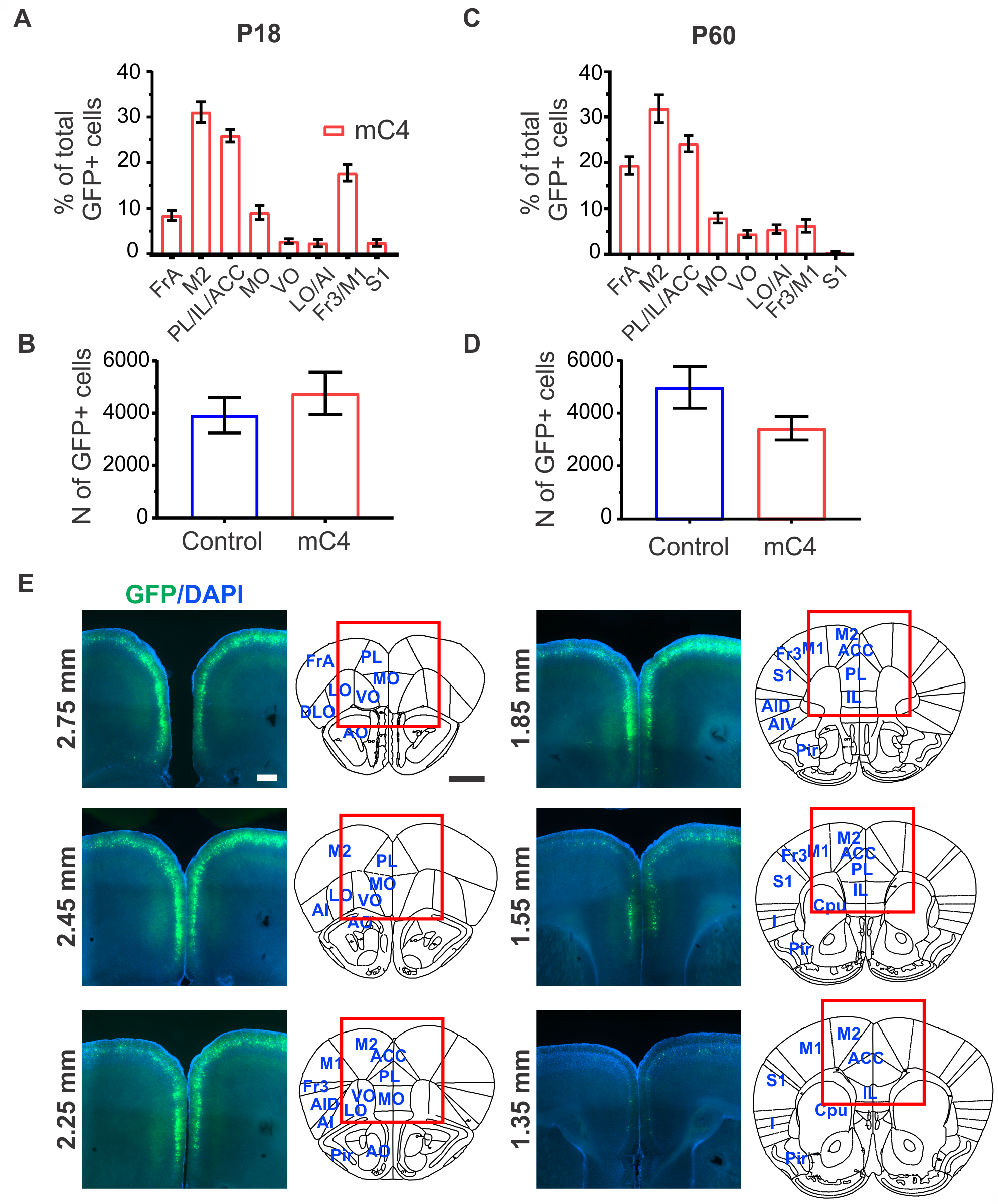

Supplement: S11 Fig — (A) Percentage of GFP+ cells per area in juvenile (P18) mC4 mice. N = 21 mC4 mice. (B) Total number of GFP+ cells for juvenile mice for control and mC4. N = 36 mice (15 control and 21 mC4 mice). (C) Percentage of GFP+ cells per area in adult mC4 mice. N = 20 mC4 mice. (D) Total number of GFP-positive cells per area for adult mice (P60) for control and mC4. N = 42 mice (22 control and 20 mC4). (E) Representative sections showing rostro-caudal extent of transfections in the frontal cortex. Images in left panels are zoomed areas from the right panels (red square). Black numbers: Bregma coordinates. Left panel scale bar = 0.5 mm. Right panel scale bar = 1 mm. Mean ± SEM. For underlying data, see https://osf.io/7em3s/?view_only=0e7ffde4ebd344dc83af83b5a605c451. ACC, anterior cingulate cortex; AI, anterior insular cortex; AO, anterior olfactory nucleus; Cpu, caudate-putamen; Fr3, frontal cortex area 3; FrA, frontal association cortex; GFP, green fluorescent protein; IL, infralimbic cortex; L, layer; LO, lateral orbitofrontal cortex; M1, primary motor cortex; M2, supplementary motor cortex; mC4, mouse C4; MO, medial orbitofrontal cortex; P, postnatal day; Pir, piriform cortex; PL, prelimbic cortex; S1, primary somatosensory cortex; VO, ventral orbitofrontal cortex. (TIF) [file pbio.3000604.s011.tif]

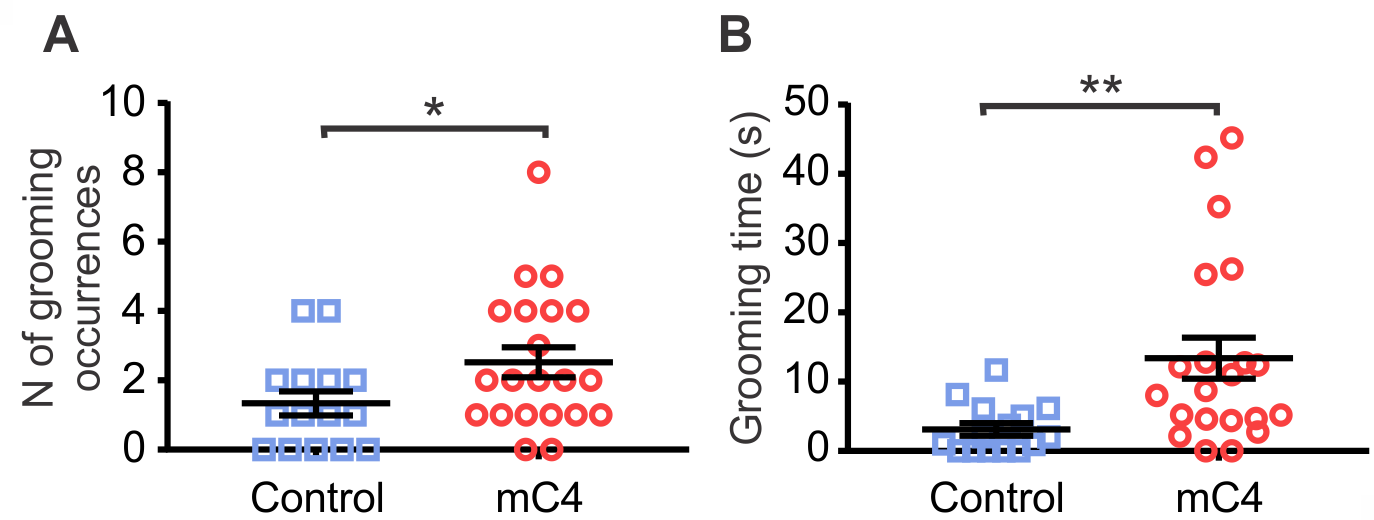

Supplement: S12 Fig — (A) mC4 mice had a greater number of grooming occurrences during the MI1 task. t test with Welch’s correction. *p < 0.05. (B) Average time per each grooming occurrence was longer for mC4 mice compared to controls in the MI1 task. t test with Welch’s correction. **p < 0.01. (A-B) N = 36 mice (15 control and 21 mC4). Mean ± SEM. For underlying data, see https://osf.io/7em3s/?view_only=0e7ffde4ebd344dc83af83b5a605c451. M2, supplementary motor cortex; mC4, mouse C4; MI1, maternal interaction 1. (TIF) [file pbio.3000604.s012.tif]

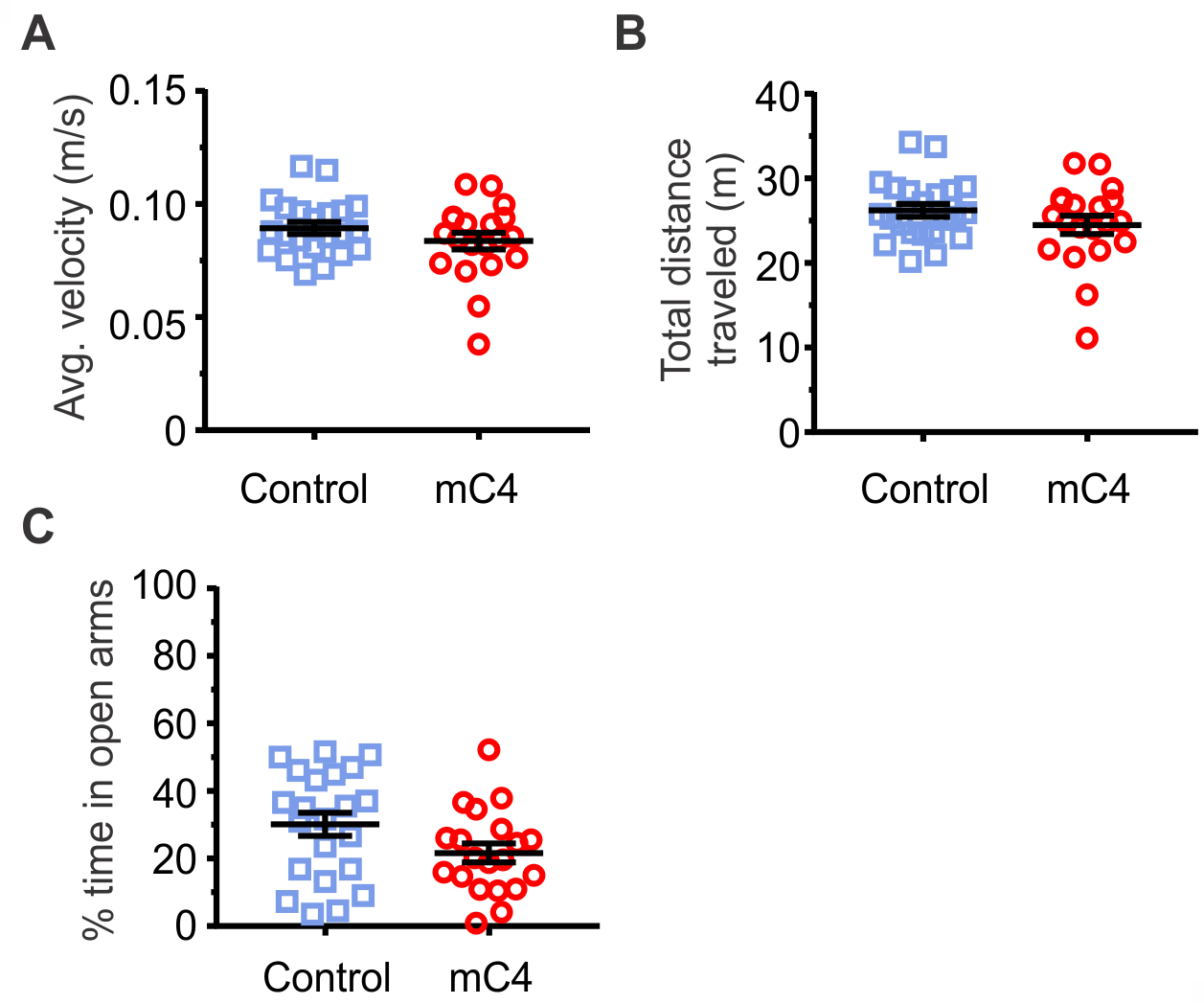

Supplement: S13 Fig — (A) Control and mC4 mice (P60) had a similar average velocity in the OF task. t test. p = 0.2141. (B) Control and mC4 mice traveled similar total distances in the OF task. t test. p = 0.1986. (C) Control and mC4 mice spent a similar amount of time exploring the open arms of the EZM task. t test. p = 0.0651. (A-C) N = 42 mice (22 control and 20 mC4 mice). Mean ± SEM. For underlying data, see https://osf.io/7em3s/?view_only=0e7ffde4ebd344dc83af83b5a605c451. EZM, elevated-zero maze; mC4, mouse C4; OF, open field; P, postnatal day. (TIF) [file pbio.3000604.s013.tif]
